# Resolution of a Configurationally Stable Hetero[4]helicene

**DOI:** 10.3390/molecules27041160

**Published:** 2022-02-09

**Authors:** Michela Lupi, Martina Onori, Stefano Menichetti, Sergio Abbate, Giovanna Longhi, Caterina Viglianisi

**Affiliations:** 1 Department of Chemistry “Ugo Schiff” (DICUS), University of Florence, Via della Lastruccia 13, Sesto Fiorentino (FI), 50019 Florence, Italy; michela.lupi@unifi.it (M.L.); martinaonori@gmail.com (M.O.); stefano.menichetti@unifi.it (S.M.); 2 Department of Molecular and Translational Medicine (DMMT), University of Brescia, V.le Europa 11 Brescia (BS), 25121 Brescia, Italy; sergio.abbate@unibs.it (S.A.); giovanna.longhi@unibs.it (G.L.)

**Keywords:** heterohelicene, chirality, resolution, enantiomers, chiroptical, screw-shaped compounds

## Abstract

We have developed an efficient chemical resolution of racemic hydroxy substituted dithia-aza[4]helicenes (DTA[4]H) **1(OH)** using enantiopure acids as resolving agents. The better diastereomeric separation was achieved on esters prepared with (1*S*)-(−)-camphanic acid. Subsequent simple manipulations produced highly optically pure (≥ 99% enantiomeric excess) (*P*) and (*M*)-**1(OH)** in good yields. The role of the position where the chiral auxiliary is inserted (*cape*- vs. *bay-zone*) and the structure of the enantiopure acid used on successful resolution are discussed.

## 1. Introduction

Chirality is one of the most crucial assets of nature and is of paramount importance in several areas of science, technology and medicine. Molecular chirality has been recognized for a long time and has provided guidance in the design of drugs and functional materials. Furthermore, a smart combination of chiral phenomena and supramolecular chemistry resulted in an emerging interdisciplinary field called supramolecular chirality [1].

**Figure 1:**
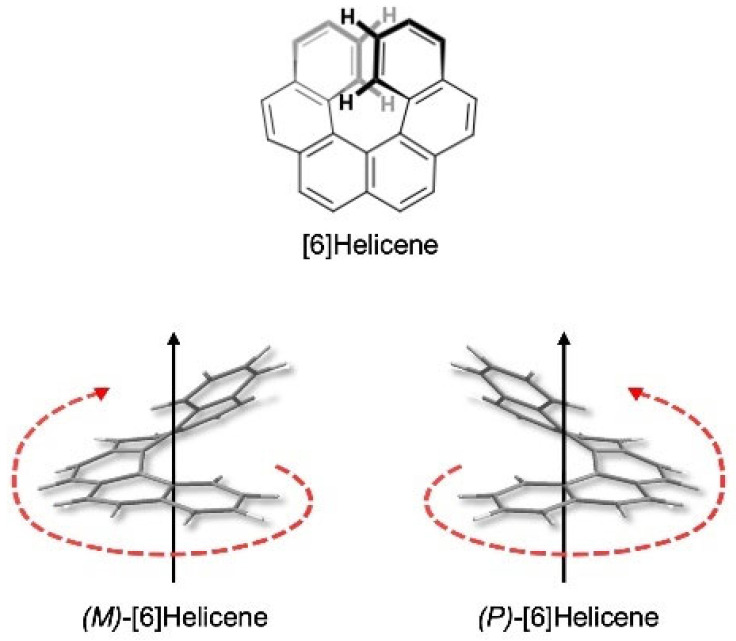
*M* and *P* enantiomers for a [6]carbohelicene.

Helicenes are compounds with a screw-shaped skeleton formed by ortho-condensed (hetero)aromatic rings with a non-planar structure due to the steric superimposition of terminal rings or/and the substituents on these rings [2], which force the molecule to adopt a helical conformation (Figure 1). This important class of axially chiral compounds has a barrier of interconversion between *M* and *P* enantiomers increasing with the increase of the ring number forming the helicene backbone.

Circular dichroism (CD) and circularly polarized luminescence (CPL) are just a few of the chiroptical proprieties that make helicenes valuable in potential applications such as advanced optical information storage, circularly polarized organic light-emitting diodes (CP-OLEDs), circularly polarized light detecting organic field effect transistors (CP-OFETs), chirality-induced spin selectivity (CISS) devices and stereoselective sensing chiroptical probes in biological processes [3,4,5,6,7,8,9,10,11,12,13,14].

Recently, increased attention has focused on the binding of small molecules to specific DNA structures to inhibit the biological functions in which these structures participate. Indeed, helicenes enantioselectivity offered a way to rationally design Z-DNA-depending inhibitors of biological functions [11].

Over the years, a variety of heterohelicenes and helical-shaped molecules, containing nitrogen, oxygen, sulfur, and other hetero-elements, have been synthesized and their unique properties studied. Great effort has been devoted to the setting of new simple and multi-gram synthetic procedures that allow for the isolation of helicenes in enantiopure form as required for various practical applications, including chirogenesis [7,15,16].

**Scheme 1:**
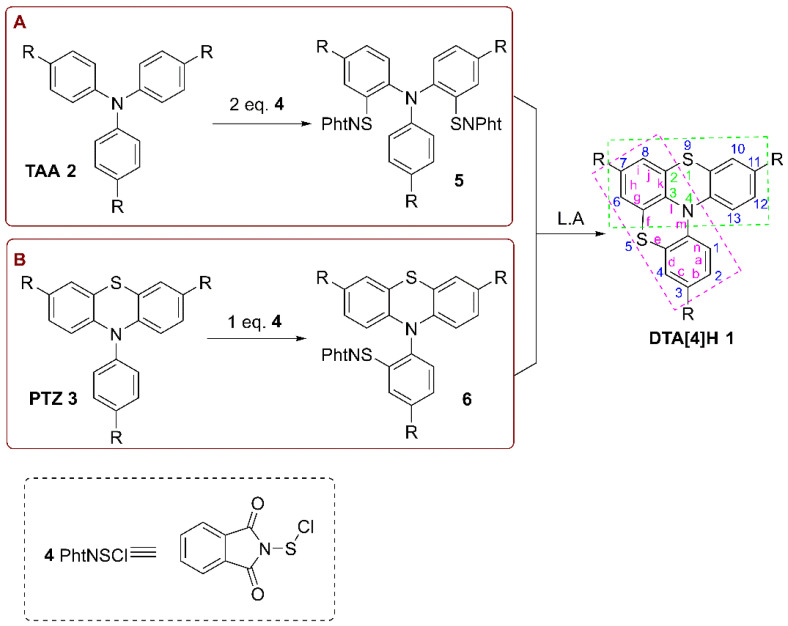
Synthetic pathways (**A** or **B**) to dithia-aza[4]helicenes (DTA[4]H) **1**.

Dithia-aza[4]helicenes (DTA[4]H) **1** (Scheme 1) can be described as bis-phenothiazines with an aryl ring and a nitrogen atom in common forced into a helical shaped structure by the four long carbon–sulfur bonds. These [1,4]benzothiazino[2,3,4-*kl*]phenothiazines represent one of the attractive rare examples of geometrically stable [4]helicenes with racemization barriers higher than those measured for all carbon [5]helicenes.

DTA[4]H are obtained [17,18,19,20,21], as racemic mixture, from properly substituted triarylamines (TAA) **2** or *N*-aryl phenothiazines (PTZ) **3** (Scheme 1, pathway A and B respectively), through regioselective sulfenylation(s) with two or one equivalents of phthalimidesulfenyl chloride (PhtNSCl (**4**), Pht = phthaloyl). Reacting the resulting sulfenylated derivatives **5** or **6** with a Lewis Acid (L.A.), typically BF_3_OEt_2_ or AlCl_3_, causes two or one electrophilic intramolecular cyclization with formation of helicenes **1**.

**Scheme 2:**
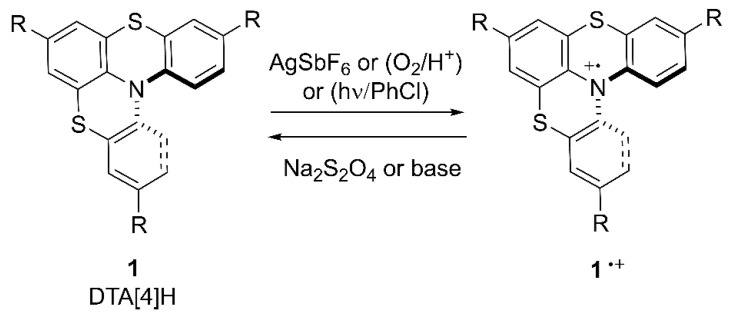
Red-ox behavior of DTA[4]H **1**.

Along with their peculiar helical-shaped structure, derivatives **1** show a very good one-electron donor ability, being easily, and reversibly, oxidized to the corresponding exceptionally stable crystalline chiral radical cations **1^•+^** [18,21] (Scheme 2). We have also demonstrated that the oxidative process is extremely sensitive to the medium, and under acidic conditions, molecular oxygen becomes an efficient single electron transfer (SET) oxidant, giving rise to the formation of **1^•+^**. Furthermore, radical cations **1^•+^** can be generated also via irradiation at 240–400 nm of helicenes in the presence of PhCl [21] (Scheme 2).

Indeed, we have also prepared, via ring-opening metathesis polymerization, dithia-aza[4]helicene functionalized polynorbornenes showing a pH depending reversible redox behavior as a new class of tunable material reversibly switchable by pH-triggered redox processes [22].

The availability of differently functionalized enantiomerically pure helicenes, avoiding the limitation associated with chiral HPLC resolution [17,23] is mandatory for the development of the appealing applications of these peculiar systems [21,22,24]. Therefore, in recent years we tried to set off regio-, stereo- and enantioselective synthetic approaches for the preparations of **1**.

Actually, the synthetic procedure depicted in Scheme 1, while failing to control the absolute stereochemistry of the process, allowed for the control of the regiochemistry during ring closure as well as the possibility of inserting different substituents in specific positions. Thus, we have studied the chemical resolutions of **1** using the classical temporary insertion of chiral auxiliaries.

**Scheme 3:**
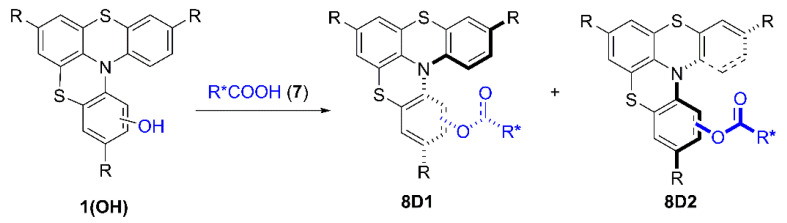
Esterification with enantiopure acids **7**.

The DTA[4]H **1** derivatives requested for the above described applications require the insertion, as an anchoring unit, of a hydroxyl group in different positions of the helical backbone. Thus, we decided to take advantage of these phenolic groups for the introduction of chiral auxiliaries through esterification with enantiopure acids **7** and in order to verify whether the diastereoisomeric mixture of esters **8** obtained can be separated. Herein we report how the helicene topology and chiral auxiliary structure could be matched to allow the resolution of phenolic DTA[4]H **1(OH)** (Scheme 3).

## 2. Results and Discussion

Selected DTA[4]H **1** can be resolved through HPLC in the chiral stationary phase as we previously reported [17,23]; however, this method is unsuitable to obtain enantiopure DTA[4]H in multigram scale. Instead, the diastereomeric process-based resolution has advantages in the the viewpoint of cost, generality and the amount of enantiopure products achieved. Thus, we decided to study the insertion of chiral auxiliaries, for example through esterification reactions, to verify whether the mixture of diastereomers obtained can be separated by flash chromatography allowing isolation of pure *M* and *P* DTA[4]H in relevant quantity.

**Scheme 4:**
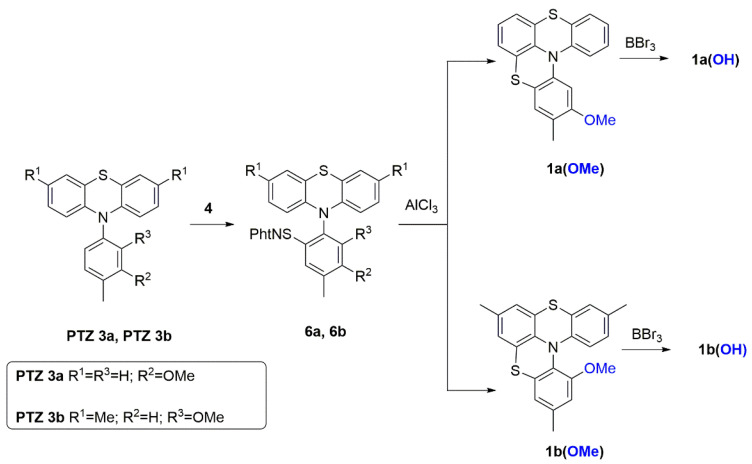
Synthesis of hydroxy substituted helicenes **1a(OH)** and **1b(OH)**.

**Scheme 5:**
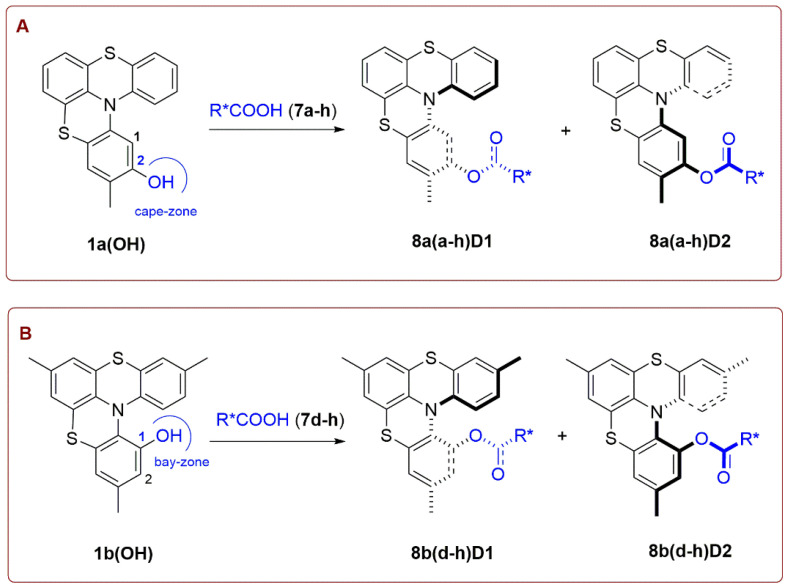
Esterification of helicenes **1a(OH)** and **1b(OH)**, panels (**A**) and (**B**), respectively, with enantiopure acids **7**.

We have demonstrated that *N*-arylphenothiazines PTZ **3** are suitable substrates for the synthesis of unsymmetrically hydroxy substituted helicenes **1(OH)** [20]. We selected helicene **1a(OH)** and **1b(OH)** (Scheme 4) to prepare diastereoisomeric esters using enantiopure acid **7** (Scheme 5).

Firstly, we planned the introduction of chiral auxiliaries in 2-hydroxy-substituted ADT[4]H **1a(OH)** presenting a hydroxyl group in the 2-position (that we indicate as *cape-zone*) of the helicene, which was relatively easy to prepare [20].

Racemic **1a(OH)** was esterified with different enantiopure acids **7a–h** yielding a diastereomeric mixture (D1+D2) of esters **8a(a–h)**. Esterification reactions were carried out in presence of diisopropylcarbodiimide (DIC) and 4-dimethylaminopyridine (DMAP) as catalysts, in dry CH_2_Cl_2_ at room temperature (Scheme 5A).

Regardless, all chiral acids **7a–h** (Scheme 5 panel A and Table 1) allowed the formation of diastereomeric esters **8a(a–h)** (Scheme 5 panel A and Table 1) in good yields, in none of these cases it was possible to separate the diastereoisomeric mixtures by flash chromatography or crystallization.

We thought, as suggested by literature data [25,26,27,28,29,30,31,32,33,34,35,36,37,38], that functionalization of the *cape-zone* position keeps the chiral auxiliaries too far from the superimposition area of the terminal aryl rings vanishing separation. Thus, we moved to helicene **1b(OH),** which allowed for the insertion of chiral auxiliaries on 1-position, *ortho* to the nitrogen atom, which we indicated as *bay-zone*, i.e., exactly in the area of terminal ring superimposition (Scheme 5B).

**Figure 2:**
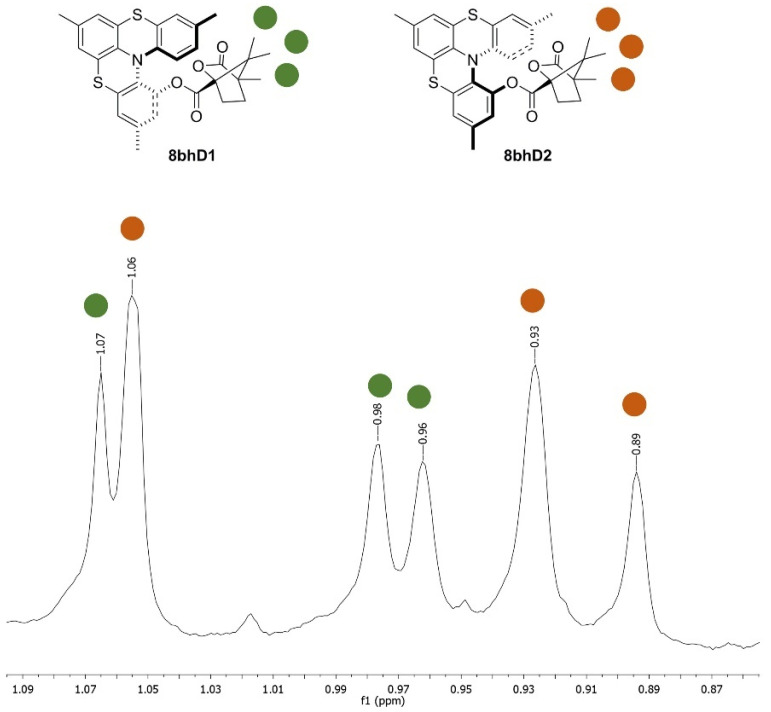
^1^H NMR spectrum of diastereomers (D1 and D2) **8bh**.

Using chiral acids **7d–h** (Scheme 5 panel B and Table 1), the corresponding diastereomeric esters **8b(d–h)** (Scheme 5 panel B and Table 1)were obtained in moderate yields generally lower than those of the corresponding esters prepared using phenol **1a(OH),** indicating, as expected, a more difficult access to the OH group of **1b(OH)** laying the *bay-zone* (Table 1). For several esters **8b** it was possible to identify the presence of the two diastereomers (D1 and D2, chromatographic elution order) by ^1^H and ^13^C NMR, and, eventually, to reveal a slightly different chromatographic behavior on TLC (Figure 2).

Despite the introduction of the chiral auxiliary in the *bay-zone* diastereoisomeric esters D1 and D2 of **8b(d–f)** were not separable by flash chromatography in spite of an accurate selection of eluent mixtures. However, esterification of **1b(OH)** with N-boc pipecolic acid **7g** allowed for the partial separation by flash chromatography on silica gel of diastereomers **8bgD1** and **8bgD2,** which were characterized by ^1^H and ^13^C NMR. Optical rotation of **8bgD1** was: ${\left[\alpha \right]}_{D}^{20}$ −157 (*c* 0.1, CH_2_Cl_2_), while for **8bgD2** was: ${\left[\alpha \right]}_{D}^{20}$ +49 (*c* 0.1, CH_2_Cl_2_). ^1^H NMR spectra show that the product **8bgD1** was isolated as single diastereomer, while the product **8bgD2** was isolated as a roughly 3:1 mixture of the two diastereomers.

Esters **8bgD1** and **8bgD2** were hydrolysed with 3 eq. of NaOH in CH_2_Cl_2_/MeOH to give enantiomeric phenols (*M*)-**1b(OH)** and (*P*)-**1b(OH)**. Phenols were analysed by HPLC in the chiral stationary phase in order to calculate the enantiomeric ratio. Chromatograms showed that product (*M*)-**1b(OH)** ${\left[\alpha \right]}_{D}^{20}$ −161 (*c* 0.1, CH_2_Cl_2_) was obtained as single enantiomer (e.e. ≥ 99%), while helicene (*P*)-**1b(OH)** ${\left[\alpha \right]}_{D}^{20}$ +75 (*c* 0.1, CH_2_Cl_2_) exhibits the enantiomeric ratio 72:28 (e.e. = 44%).

**Figure 3:**
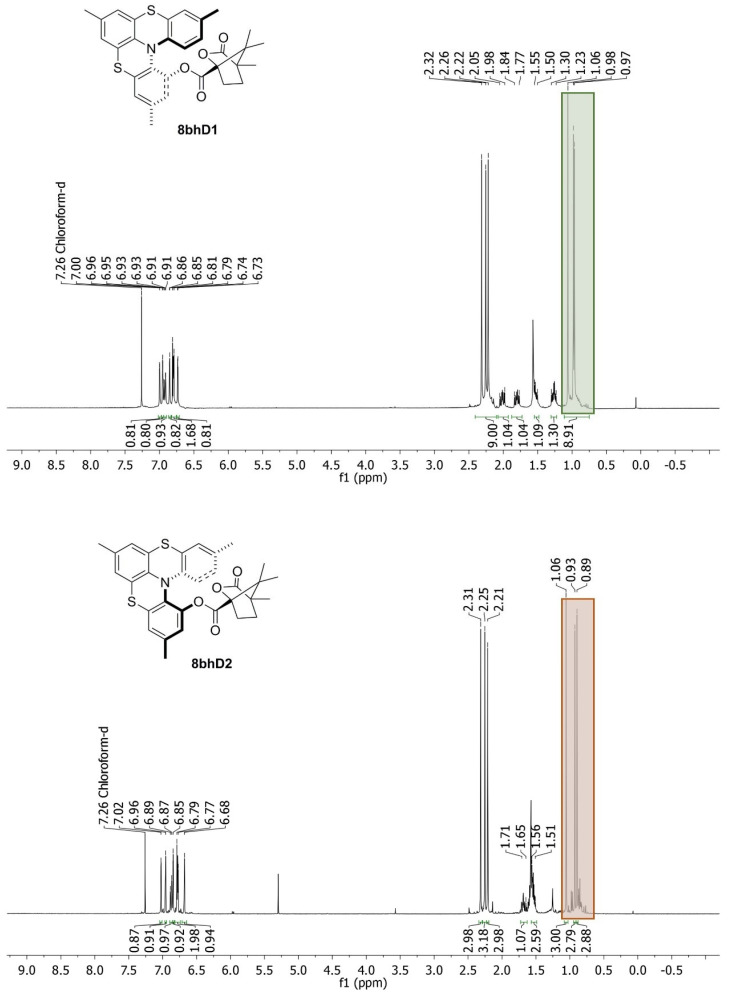
^1^H-NMR spectra of diastereomers **8bhD1** and **8bhD2**.

Esterification of **1b(OH)** with (1*S*)-(−)-camphanic acid **7h** provided, with our great satisfaction, diastereomers **8bhD1** and **8bhD2** that were successfully separated by flash chromatography and characterized by ^1^H and ^13^C NMR spectroscopy (Figure 3 and Supplementary Materials).

**Scheme 6:**
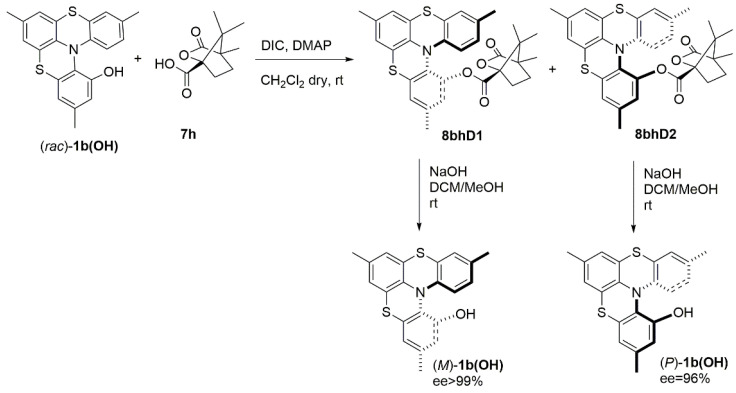
Chemical resolution of helicene (*rac*)-**1b(OH)**.

Optical rotation was measured and gave ${\left[\alpha \right]}_{D}^{20}$ −129 (*c* 0.1, CH_2_Cl_2_) for **8bhD1** and ${\left[\alpha \right]}_{D}^{20}$ + 126, (*c* 0.1, CH_2_Cl_2_) for **8bhD2**. Hydrolysis of diastereomeric esters provided helicenes (*M*)-**1b(OH)** and (*P*)-**1b(OH)**, respectively. HPLC analysis with a chiral stationary phase showed that the first eluted product, (*P*)-**1b(OH)** ${\left[\alpha \right]}_{D}^{20}$ +166 (*c* 0.1, CH_2_Cl_2_), exhibits an enantiomeric ratio = 98:2 (e.e. = 96%), while the second eluted product, (*M*)-**1b**(OH) ${\left[\alpha \right]}_{D}^{20}$ −167 (*c* 0.1, CH_2_Cl_2_), was obtained as a single enantiomer (e.e. ≥ 99%), (Scheme 6).

The assignment of the absolute configuration of DTA[4]H **1** derivatives has been established as *P*-(+) and *M*-(−), which is typical for helicene systems; opposite assignment is quite unusual, as we have established in ref [17,19]. In this work the absolute configuration of **1b(OH)** was validated by comparison of the electronic circular dichroism (ECD) spectra of the two optical enantiomers-(+)-**1b(OH)** and (−)-**1b(OH)**, assigned to (*P*)-**1b(OH)** and (*M*)-**1b(OH)**, with the calculated spectrum of the *M* structure.

**Figure 4:**
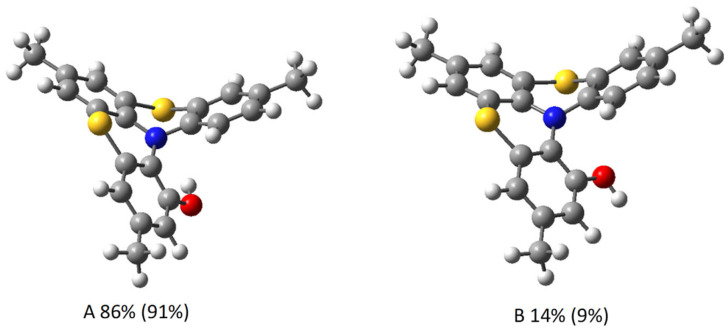
Optimized 3D-structures for the two possible conformers of (*M*)-**1b(OH)**. Percent population factors calculated at M06/cc-pvtz, iefpcm level; in parenthesis population factors calculated at M062X/cc-pvtz, iefpcm level.

In order to assign the configuration, DFT and TD-DFT calculations have been conducted with the Gaussian16 package [39]. Two orientations are possible for the hydroxyl-group; the two optimized structures in the *M* configuration are reported in Figure 4 with their Boltzmann populations. Two functionals have been considered; the two differing in the amount of the exact exchange included M06 with 27% HF exchange and M06-2X with 54% HF exchange [40]. The solvent has been treated at the iefpcm level. Structural results are similar for the two functionals.

CD and absorption spectra have been calculated at the same level of theory, a constant Gaussian 0.2 eV bandwidth was applied to each transition. The experimental CD and absorption spectra have been recorded for the two enantiomers in 4.2 mM dichloromethane solution in a 0.1 mm quartz cuvette using a JASCO-815SE instrument.

**Figure 5:**
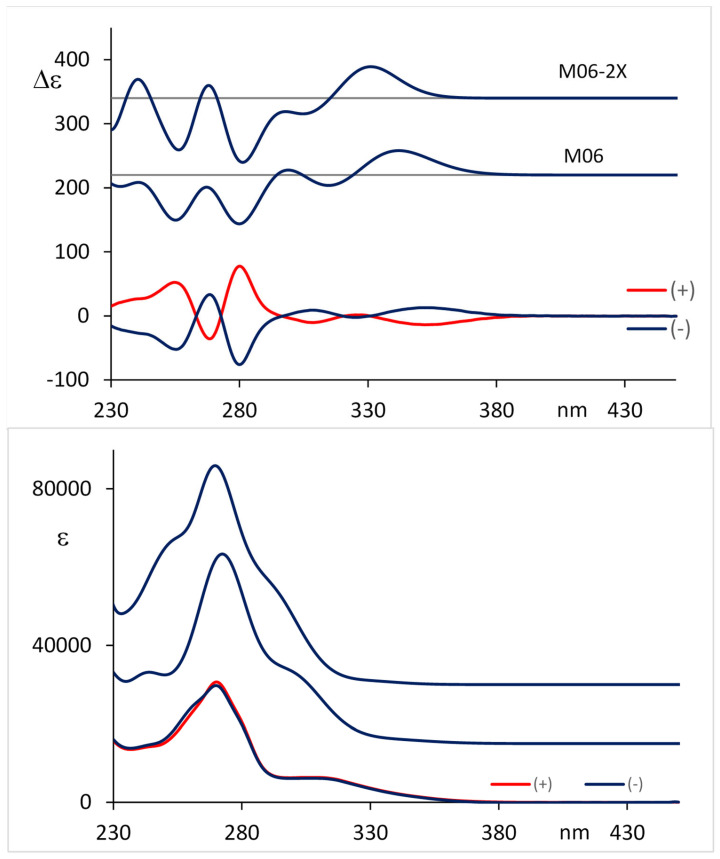
CD (top) and absorption (bottom) experimental and calculated spectra with two choices for the DFT functional (see text); calculation performed on *M*-**1b(OH)**. The calculated spectra are Boltzmann weighed averages of the two conformers A and B.

The comparison of experimental data with calculations are presented in Figure 5. In order to compare with data, +4 nm shift has been applied to the results obtained with M06, +26 nm with M06-2X; calculation of similarity index between experimental and calculated spectra suggested the best shift for the best correspondence, as reported in the Supplementary Materials paragraph. It is also shown that the two conformers give very similar spectra, while in Figure 5 the Boltzmann weighed average is presented. Both functionals permit confirmation of the configuration as *M*-(-) (correspondingly *P*-(+)). This conclusion agrees with what was obtained for the parent molecule triarylamine hetero[4]helicene examined in reference [19].

Overall, our results confirm that, on chemical resolution of helicenes, the position where the chiral auxiliaries are inserted is extremely important, being the *bay-zone* that allows for the higher effect on enantiomeric discrimination. At the same time we have confirmed previous studies reporting chromatographic resolutions of [7]carbo- and [7]hetero-helicenes by means of tetra- and monocamphanate esters [25,26,27,28,29,30,31]. In each of these cases, the (1*S*)-camphanate of the (*P*)-helicenol moves more slowly upon chromatography on silica gel than the (1*S*)-camphanate of the (*M*)-helicenol [27].

## 3. Materials and Methods

^1^H and ^13^C NMR spectra were recorded with Varian Mercury Plus 400, Varian Inova 400, using CDCl_3_ as solvent. Residual CHCl_3_ at *δ* = 7.26 ppm and central line of CDCl_3_ at *δ* = 77.16 ppm were used as the reference of ^1^H-NMR spectra and ^13^C NMR spectra, respectively. FT-IR spectra were recorded with a Spectrum Two FT-IR Spectrometer. ESI-MS spectra were recorded with a JEOL MStation JMS700. Melting points were measured with a Stuart SMP50 Automatic Melting Point Apparatus. Optical rotation measurements were performed on a JASCO DIP-370 polarimeter (JASCO, Easton, MD, USA) and the specific rotation of compounds was reported [41].

All the reactions were monitored by TLC on commercially available precoated plates (silica gel 60 F 254) and the products were visualized with acidic vanillin solution. Silica gel 60 (230–400 mesh) was used for column chromatography. Dry solvents were obtained by The PureSolv Micro Solvent Purification System. Chloroform was washed with water several times and stored over calcium chloride. Pyridine and TEA were freshly distilled from KOH. Phthalimide sulfenyl chloride was prepared from the corresponding disulfide (purchased from Chemper snc) as reported elsewhere. Helicenes **1a** and **1b** were described elsewhere [17].

General Procedure for the synthesis of diastereomeric esters from **1** by Steglich esterification: To a solution of **1** in dry CH_2_Cl_2_ (roughly 0.03–0.04 M), the enantiopure acid **7** (1.2 eq), DMAP (0.1 eq) and DIC (1–1.2 eq) were added at 0 °C. The solution was stirred at room temperature under a nitrogen atmosphere for 2–29 h, then was diluted with CH_2_Cl_2_ (60 mL), washed with a saturated solution of NH_4_Cl (2 × 40 mL), with a saturated solution of NaHCO_3_ (3 × 40) then with NH_4_Cl (3 × 40 mL). The organic layer was dried over Na_2_SO_4_, filtered and evaporated under reduced pressure. The crude was purified by flash chromatography on silica gel.

Diastereoisomers **8aaD1** and **8aaD2**. (*M/P*)-3-methyl[1,4]benzothiazino[2,3,4-*kl*]phenothiazine-2-yl (*S*)-perillate. Following the general *Steglich esterification* procedure from **1a(OH)** (60 mg, 0.18 mmol) and (*S*)-(−)-perillic acid **7a** (36 mg, 0.22 mmol), kept for 22 h at rt. The crude was purified by flash chromatography on silica gel (petroleum ether/ CH_2_Cl_2_ 1:3, *Rf* 0.86) to afford the mixture of the two diastereomeric compounds **8aaD1** and **8aaD2** (52 mg, 60% yield) as a white solid (mp 105–115 °C). ^1^H NMR (400 MHz, CDCl_3_)* δ: 1.45- 1.55 (m, 2H), 1.76 (s, 6H), 1.89–1.95 (m, 2H), 2.12 (s, 6H), 2.16–2.41 (m, 8H), 2.51–2.59 (m, 2H), 4.74 (bs, 2H), 4.78 (bs, 2H), 6.89 (bs, 2H) 6.92–7.05 (m, 8H), 7.06 (bs, 2H), 7.12–7.25 (m, 8H) ppm. ^13^C NMR (100 MHz, CDCl_3_)* δ: 15.8, 20.9, 24.76, 24.80, 27.1, 31.4, 40.1, 108.63, 108, 64, 110.0, 110.6, 114.7, 120.6, 124.1, 124.8, 125.0, 125.7, 125.75, 125.83, 126.0, 127.0, 127.3, 127.7, 128.0, 129.3, 129.5, 139.5, 141.2, 141.8, 142.5, 148.7, 149.2, 165.2, 165.3 ppm (34 signals for 58 different carbons). Elem. Anal. for C_29_H_25_NO_2_S_2_: Calcd. C 72.02, H 5.21, N 2.90; found C 71.80; H 5.21, N 2.89. *Et_3_N was added to neutralize CHCl_3_ acidity.

Diastereoisomers **8abD1** and **8abD2**. (*M/P*)-3-methyl[1,4]benzothiazino[2,3,4-*kl*]phenothiazine-2-yl (1*S*)-10-camphorsulfonate. To a solution of **1a(OH)** (60 mg; 0.18 mmol) and TEA (22 mg, 0.22 mmol) in 4 mL of dry CH_2_Cl_2_, (1*S*)-(+)-10-camphorsulfonyl chloride **7b** (51 mg, 0.20 mmol) is added at 0 °C. After 10 min the solution was allowed to warm at room temperature and was stirred for 18 h under a nitrogen atmosphere. The mixture was diluted with AcOEt (25 mL) and washed with water (3 × 20 mL). The organic layer was dried over Na_2_SO_4_, filtered, and then evaporated under reduced pressure. The crude was purified by flash chromatography on silica gel (petroleum ether/AcOEt: 5/1, *Rf* 0.38) to afford the mixture of the two diastereomeric compounds **8abD1** and **8abD2** (55 mg, 56% yield) as a white solid (mp 190–195 °C). ^1^H NMR (400 MHz, CDCl_3_) δ: 0.87 (s, 3H), 0.88 (s, 3H), 1.11 (s, 6H), 1.40–1.47 (m, 2H), 1.64–1.72 (m, 2H), 1.92–1.98 (m, 2H), 2.00–2.13 (m, 4H), 2.31 (s, 3H), 2.32 (s, 3H), 2.36–2.57 (m, 4H), 3.13–3.18 (m, 2H), 3.73–3.78 (m, 2H), 6.93–7.25 (m, 18H) ppm. ^13^C NMR (100 MHz, CDCl_3_) δ: 11.6, 16.4, 16.5, 19.8, 20.02, 20.03, 25.3, 26.9, 27.0, 42.5, 43.0, 43.1, 46.3, 48.01, 48.03, 48.4, 48.6, 58.19, 58.24, 114.7, 114.8, 120.45, 120.50, 125.1, 125.2, 125.4, 125.5, 125.7, 125.8, 125.9, 126.0, 126.1, 126.2, 127.1, 127.8, 127.9, 128.11, 128.13, 128.2, 128.4, 130.0, 130.1, 139.2, 141.29, 141.32, 142.26, 142.29, 147.0, 147.1, 213.8, 213.9 ppm. Elem. Anal. for C_29_H_27_NO_4_S_3_: Calcd. C 63.36, H 4.95, N 2.55; found C 63.38, H 4.95, N 2. 54.

Diastereoisomers **8acD1** and **8acD2**. (*M/P*)-3-methyl[1,4]benzothiazino[2,3,4-*kl*]phenothiazine-2-yl o,o’-dibenzoyl-L-tartrate. Following the general procedure from **1a(OH)** (85 mg, 0.25 mmol) and (-)-o,o’-dibenzoyl-L-tartaric acid mono(dimethyl amide) **7c** (117 mg, 0.30 mmol), kept for 2 h at room temperature. The crude was purified by flash chromatography on silica gel (AcOEt/CH_2_Cl_2_ 1/20, *Rf* 0.65) to afford the mixture of the two diastereomeric compounds **8acD1** and **8acD2** (99 mg, 56% yield) as a white solid (mp 102–106 °C). ^1^H NMR (400 MHz, CDCl_3_) δ: 2.09 (s, 3H), 2.10 (s, 3H), 2.92 (s, 3H), 2.93 (s, 3H), 3.17 (s, 3H), 3.30 (s, 3H), 6.16 (d, 1H, *J* = 4.8 Hz), 6.20 (d, 1H, *J* = 5.2 Hz), 6.31 (d, 1H, *J* = 4.8 Hz), 6.33 (d, 1H, *J* = 5.2 Hz), 6.83–7.20 (m, 18H), 7.44–7.54 (m, 8H), 7.54–7.58 (m, 4H), 7.99–8.08 (m, 8H) ppm. ^13^C NMR (100 MHz, CDCl_3_) δ: 15.61, 15.63, 36.27, 36.31, 37.2, 69.5, 69.6, 70.9, 113.8, 114.0, 120.35, 120.41, 124.8, 124.9, 125.0, 125.1, 125.5, 125.6, 125.72, 125.74, 125.86, 125.92, 126.75, 126.84, 126.9, 127.6, 127.7, 127.9, 128.0, 128.35, 128.43, 128.52, 128.53, 128.58, 128.59, 128.60, 129.58, 129.59, 130.0, 130.09, 130.12, 130.15 133.82, 133.86, 139.12, 139.13, 141.1, 141.2, 142.1, 142.3, 148.09, 148.10, 165.0, 165.1, 165.3, 165.43, 165.44, 165.45, 165.50 ppm (59 signals for 78 carbons). IR (ATR solid) n: 3063, 2929, 1763, 1724, 1664, 1477, 1432, 1240 cm^−1^. Elem. Anal. for C_39_H_30_N_2_O_7_S_2_: Calcd. C 66.65, H 4.30, N 3.99; found C 66.61, H 4.28, N 4.00.

Diastereoisomers **8adD1** and **8adD2**. (*M/P*)-3-methyl[1,4]benzothiazino[2,3,4-*kl*]phenothiazine-2-yl (1*S*)-ketopinate. Following the general *Steglich esterification* procedure from **1a(OH)** (60 mg, 0.18 mmol) and (1*S*)-(+)-ketopinic acid **7d** (56 mg, 0.31 mmol), kept for 29 h at room temperature. The crude was purified by flash chromatography on silica gel (petroleum ether/CH_2_Cl_2_ 1/3, *Rf* 0.45) to afford the mixture of the two diastereomeric compounds **8adD1** and **8adD2** (71 mg, 79% yield) as a white solid (mp 130–133 °C). ^1^H NMR (400 MHz, CDCl_3_)* δ: 1.14 (s, 6H), 1.21 (s, 3H), 1.22 (s, 3H), 1.42–1.48 (m, 2H), 1.88–1.97 (m, 3H), 2.01–2.10 (m, 3H), 2.13–2.15 (m, 2H), 2.18 (s, 3H), 2.19 (s, 3H), 2.40–2.48 (m, 2H), 2.54–2.60 (m, 2H), 6.85 (s, 1H), 6.87 (s, 1H), 6.94–7.06 (m, 10H), 7.12–7.23 (m, 6H) ppm. ^13^C NMR (100 MHz, CDCl_3_)* δ: 16.2, 20.0, 21.50, 21.52, 26.49, 26.52, 26.7, 26.8, 44.02, 44.58, 44.61, 49.51, 49.56, 68.20, 68.24, 110.6, 114.70, 114.72, 120.41, 120.44, 124.6, 124.8, 124.9, 125.0, 125.70, 125.72, 125.8, 126.1, 126.8, 127.2, 127.3, 127.75, 127.82, 128.1, 129.59, 129.61, 139.4, 140.9, 141.0, 142.66, 142.71, 148.8, 168.1, 210.3 ppm (44 signals for 58 different carbons). Elem. Anal. for C_29_H_25_NO_3_S_2_: Calcd. C 69.71, H 5.04, N 2.80; found: C 69.73, H 5.01, N 2.77. *Et_3_N was added to neutralize CHCl_3_ acidity.

Diastereoisomers **8aeD1** and **8aeD2**. (*M/P*)-3-methyl[1,4]benzothiazino[2,3,4-*kl*]phenothiazine-2-yl (*S*)-2-(6-methoxy-2-naphthyl) propionate. Following the general *Steglich esterification* procedure from **1a(OH)** (60 mg, 0.18 mmol) and (*S*)-(+)-2-(6-methoxy-2-naphthyl) propionic acid **7e** (50 mg, 0.22 mmol), kept for 20 h at room temperature. The crude was purified by flash chromatography on silica gel (petroleum ether/CH_2_Cl_2_ 1/3, *Rf* 0.80) to afford the mixture of the two diastereomeric compounds **8aeD1** and **8aeD2** (94 mg, 94% yield) as an orange solid (mp 132–136 °C). ^1^H NMR (400 MHz, CDCl_3_)* δ: 1.39 (d, 6H, *J* = 6.5 Hz), 1.77 (s, 3H), 1.79 (s, 3H), 3.39 (s, 6H), 3.84 (q, 1H, *J* = 6.6 Hz), 4.12 (q, 1H, *J* = 6.7 Hz), 6.81 (bs, 2H), 6.91–7.05 (m, 10H), 7.10–7.20 (m, 10H), 7.44–7.47 (m, 2H), 7.67–7.73 (m, 6H) ppm. ^13^C NMR (100 MHz, CDCl_3_)* δ: 15.46, 15.48, 18.5, 18.6, 20.6, 21.2, 22.7, 23.6, 42.3, 45.56, 45.59, 55.4, 105.7, 108.6, 110.6, 114.4, 114.5, 119.3, 120.5, 124.3, 124.8, 125.0, 125.7, 125.8, 125.96, 126.01, 126.3, 126.4, 126.9, 127.1, 127.2, 127.4, 127.7, 127.8, 127.98, 128.01, 129.0, 129.4, 129.5, 133.9, 134.95, 134.99, 139.4, 141.0, 142.50, 142.54, 148.9, 157.9, 172.7 ppm (49 signals for 66 different carbons). Elem. Anal. for C_33_H_25_NO_3_S_2_: Calcd. C 72.37, H 4.60, N 2.56; found C 72.29; H 4.58, N 2.57. *Et_3_N was added to neutralize CHCl_3_ acidity.

Diastereoisomers **8afD1** and **8afD2**. (*M/P*)-3-methyl[1,4]benzothiazino[2,3,4-*kl*]phenothiazine-2-yl mono-(1*R*)-menthylphthalate. Following the general procedure from **1a(OH)** (70 mg, 0.21 mmol) and (-)mono(1*R*)-menthylphthalate **7f** (76 mg, 0.25 mmol), kept for 24 h at room temperature. The crude was purified by flash chromatography on silica gel (petroleum ether/ CH_2_Cl_2_ 2/1, *Rf* 0.78)) to afford the mixture of the two diastereomeric compounds **8afD1** and **8afD2** (70 mg, 54% yield) as a white solid (mp 140.8–146.8 °C). ^1^HNMR (400 MHz, CDCl_3_) δ: 0.73–0.69 (m, 6H), 0.83–0.89 (m, 14H), 0.97–1.16 (m, 4H), 1.36–1.51 (m, 4H), 1.64–1.71 (m, 4H), 1.85–1.98 (m, 2H), 2.05–2.11 (m, 2H), 2.21 (s, 3H), 2.22 (s, 3H), 4.82–4.89 (m, 2H), 6.93–7.00 (m, 6H), 7.03 (dd, 2H, *J* = 1.2 Hz, *J* = 7.5 Hz), 7.08 (bs, 1H), 7.09 (bs, 1H), 7.11–7.16 (m, 2H), 7.18 (d, 2H, *J* = 7.7 Hz), 7.24–7.29 (m, 2H), 7.54–7.60 (m, 4H), 7.68–7.73 (m, 2H), 7.85–7.90 (m, 2H) ppm. ^13^C NMR (100 MHz, CDCl_3_) δ: 15.19, 16.0, 16.3, 16.5, 20.89, 20.93, 22.15, 22.18, 23.5, 23.6, 26.3, 26.4, 31.55, 31.57, 34.37, 34.41, 40.6, 40.7, 47.23, 47.25, 75.95, 75.02, 114.47, 114.49, 120.60, 120.64, 124.5, 124.6, 124.9, 125.0, 125.71, 125.75, 125.77, 125.79, 126.07, 126.08, 126.95, 126.99, 127.33, 127.34, 127.71, 127.75, 127.98, 128.00, 128.8, 129.0, 129.51, 129.52, 129.56, 129.60, 130.6, 130.89, 130.94, 131.0, 131.8, 131.9, 133.5, 133.7, 139.5, 141.2, 142.50, 142.52, 148.97, 148.99, 165.3, 165.4, 166.8, 166.9 ppm (68 signals for 74 different carbons). IR (ATR solid) n: 2953, 2923, 2867, 1749, 1715, 1477, 1431, 1276, 1236 cm^−1^. Elem. Anal. for C_37_H_35_NO_4_S_2_: Calcd. C 71.47, H 5.67, N 2.25; found C 71.36, H 5.65, N 2.26.

Diastereoisomers **8agD1** and **8agD2**. (*M/P*)-3-methyl[1,4]benzothiazino[2,3,4-*kl*]phenothiazine-2-yl (S)-N-Boc pipecolinate. Following the general procedure from **1a(OH)** (61 mg, 0.18 mmol) and (S)-N-Boc pipecolic acid **7g** (50 mg, 0.22 mmol) kept for 22 h at room temperature. The crude was purified by flash chromatography (CH_2_Cl_2_, *Rf* 0.51) on silica gel to afford the mixture of the two diastereomeric compounds **8agD1** and **8agD2** (70 mg, 69%yield) as a white solid (mp 106–117 °C). ^1^H NMR (400 MHz, CDCl_3_)* δ: 1.37–1.44 (m, 22H), 1.65–1.80 (m, 8H), 2.13 (s, 6H), 2.29–2.33 (m, 2H), 2.93–3.09 (m, 2H), 3.89–3.95 (m, 1H), 4.04–4.09 (m, 2H), 4.92 (bs, 1H), 5.07 (bs, 1H), 7.21–6.77 (m, 18H) ppm. Elem. Anal. For C_30_H_30_N_2_O_4_S_2_: Calcd. C 65.91, H 5.53, N 5.12; found: C 65.35, H 5.31, N 4.98. *Et_3_N was added to neutralize CHCl_3_ acidity.

Diastereoisomers **8ahD1** and **8ahD2**. (*M/P*)-3-methyl[1,4]benzothiazino[2,3,4-*kl*]phenothiazine-2-yl (1*S*)-camphanate. Following a *Steglich esterification* procedure from **1a(OH)** (60 mg, 0.18 mmol) and (1*S*)-(−)-camphanic acid **7h** (43 mg, 0.22 mmol), kept for 17 h at room temperature. The crude was purified by flash chromatography on silica gel (petroleum ether/AcOEt/diethyl ether: 10:1:3, *Rf* 0.43)) to afford the mixture of the two diastereomeric compounds **8ahD1** and **8ahD2** (52 mg, 57% yield) as a white solid (mp 170 °C dec). ^1^H NMR (400 MHz, CDCl_3_)* δ: 1.04 (s, 3H), 1.06 (s, 3H), 1.11 (s, 6H), 1.13 (s, 3H), 1.14 (s, 3H), 1.69–1.77 (m, 2H), 1.92–1.99 (m, 2H), 2.11–2.19 (m, 8H), 2.45–2.54 (m, 2H), 6.84 (s, 1H), 6.86 (s, 1H), 6.93–7.00 (m, 6H), 7.02–7.08 (m, 4H), 7.12–7.21 (m, 6H) ppm. ^13^C NMR (100 MHz, CDCl_3_)* δ: 9.78, 9.81, 16.0, 16.2, 16.94, 16.96, 17.01, 17.04, 29.0, 31.2, 31.3. 53.6, 54.6, 54.97, 55.00, 90.89, 90.94, 114.16, 114.21, 120.4, 120.5, 125.0, 125.1, 125.24, 125.27, 125.6, 125.8, 125.9, 126.0, 126.1, 126.50, 126.52, 126.9, 127.0, 127.8, 128.1, 129.8, 139.3, 141.2, 141.3, 142.4, 142.5, 148.1, 148.2, 165.8, 165.9, 177.8 ppm (47 signals for 58 different carbons). Elem. Anal. for C_29_H_25_NO_4_S_2_: Calcd. C 67.55, H 4.89, N 2.72; found C 67.49, H 4.88, N 2.72. *Et_3_N was added to neutralize CHCl_3_ acidity.

Diastereoisomers **8bdD1**, **8bdD2**. (*M/P*)-1,3,7-trimethyl[1,4]benzothiazino[2,3,4-*kl*]phenothiazine-2-yl (1*S*)-ketopinate. Following procedure from **1b(OH)** (40 mg, 0.11 mmol) and (1*S*)-(+)-ketopinic acid **7d** (20 mg, 0.11 mmol), kept for 18 h at room temperature. The crude was purified by flash chromatography on silica gel (petroleum eter/CH_2_Cl_2_ 1/2, *Rf* 0.38)) to afford the mixture of the two diastereomeric compounds **8bdD1** and **8bdD2** (32 mg, 55% yield) as a white solid (mp 190–199 °C). ^1^H NMR (400 MHz, CDCl_3_) δ: 0.76 (s,3H), 1.01 (s, 3H), 1.06 (s, 3H), 1.11 (s, 3H), 1.18–1.36 (m, 5H), 1.63–1.70 (m, 1H), 1.73–1.90 (m, 4H), 1.94–1.99 (m, 2H), 2.21–2.31 (m, 18H), 2.42–2.49 (m, 2H), 6.77–6.84 (m, 7H), 6.88–7.01 (m, 7H) ppm. ^13^C NMR (100 MHz, CDCl3) δ: 19.61, 19.64, 20.51, 20.54, 20.61, 20.7, 20.78, 20.82, 20.9, 24.9, 25.3, 26.16, 26.24, 43.7, 43.8, 43.9, 44.0, 48.2, 48.5, 67.5, 67.6, 118.1, 118.6, 123.4, 123.8, 125.45, 125.55, 125.6, 125.78, 125.83, 125.9, 126.0, 126.3, 126.4, 127.0, 127.2, 127.5, 127.8, 128.3, 128.5, 129.4, 130.0, 130.7, 130.9, 133.6, 133.7, 134.8, 135.70, 135.74, 138.0, 138.1, 142.1, 142.4, 142.5, 167.2, 167.8, 210.54, 210.55 ppm (58 signals for 62 carbons). IR (ATR solid) n: 2961, 2920, 2888, 1762, 1737, 1480, 1448, 1313, 1270 cm^−1^. Elem. Anal. for C_31_H_29_NO_3_S_2_: Calcd. C 70.56, H 5.54, N 2.65; found C 70.48, H 5.55, N 2.64.

Diastereoisomers **8beD1** and **8beD2**. (*M/P*)-1,3,7-trimethyl[1,4]benzothiazino[2,3,4-*kl*]phenothiazine-2-yl (*S*)-2-(6-methoxy-2-naphthyl) propionate. Following the general procedure from **1b(OH)** (48 mg, 0.13 mmol) and (*S*)-(+)-2-(6-methoxy-2-naphthyl) propionic acid **7e** (28 mg, 0.13 mmol), kept for 12 h at room temperature. The crude was purified by flash chromatography on silica gel (petroleum ether/CH_2_Cl_2_ 1/2, *Rf* 0.74)) to afford the mixture of the two diastereomeric compounds **8beD1** and **8beD2** (43 mg, 58% yield) as a white solid (mp 250 °C dec). ^1^H NMR (400 MHz, CDCl_3_) δ: 1.23 (d, 3H, *J* = 7.2 Hz), 1.39 (d, 3H, *J* = 7.2 Hz) 2.208 (s, 3H), 2.214 (s, 3H), 2.22 (s, 3H), 2.23 (s, 3H), 2.29 (s, 3H), 2.33 (s, 3H), 3.10 (q, 1H, *J* = 7.3 Hz), 3.20 (q, 1H, *J* = 7.2 Hz), 3.91 (s, 3H), 3.92 (s, 3H), 6.52 (bs, 1H), 6.65 (bs, 1H), 6.75–6.94 (m, 10H), 6.99 (bs, 1H), 7.04 (bs, 1H), 7.09–7.17 (m, 5H), 7.27–7.30 (m, 1H), 7.39 (bs, 1H), 7.53 (bs, 1H), 7.64–7.71 (m, 4H) ppm. Elem Anal. for C_35_H_29_NO_3_S_2_: Calcd. C 73.02, H 5.08, N 2.43; found C 73.03, H 5.09, N 2.43.

Diastereoisomers **8bfD1** and **8bfD2**. (*M/P*)-1,3,7-trimethyl[1,4]benzothiazino[2,3,4-*kl*]phenothiazine-2-yl mono(1*R*)-menthylphthalate. Following the general procedure from **1b(OH)** (40 mg, 0.11 mmol) and (-)mono(1*R*)-menthylphthalate **7f** (33 mg, 0.11 mmol), kept for 18 h at room temperature. The crude was purified by flash chromatography on silica gel (petroleum ether/CH_2_Cl_2_ 1/1, *Rf* 0.63)) to afford the mixture of the two diastereomeric compounds **8bfD1** and **8bfD2** (32 mg, 45% yield) as a white solid (mp 180–190 °C). ^1^HNMR (400 MHz, CDCl_3_) δ: 0.66 (d, 3H, *J* = 6.9 Hz), 0.78–0.90 (m, 17H), 0.98–1.11 (m, 4H), 1.40–1.53 (m, 4H), 1.65–1.71 (m, 4H), 1.90–1.99 (m, 2H), 2.05–2.26 (m, 14H), 2.53 (s, 6H), 4.85–4.93 (m, 2H), 6.70–6.85 (m, 10H), 6.95–7.00 (m, 4H), 7.07 (bs, 2H), 7.26–7.30 (m, 2H), 7.44–7.54 (m, 4H) ppm. ^13^CNMR (100 MHz, CDCl_3_) δ: 16.1, 16.4, 20.5, 20.6, 20.90, 20.92, 21.02, 22.1, 22.2, 23.4, 23.5, 26.2, 26.3, 31.5, 31.6, 34.4, 34.5, 40.5, 40.7, 47.3, 47.4, 75.7, 118.45, 118.50, 123.35, 123.40, 125.7, 125.76, 125.77, 125.79, 125.9, 126.26, 126.34, 126.4, 127.0, 127.46, 127.48, 128.05, 128.12. 128.16, 128.3, 129.50, 129.53, 129.6, 129.75, 129.77, 130.15, 130.18, 130.7, 130.8, 131.4, 131.5, 133.6, 133.9, 134.1, 134.7, 134.8, 135.36, 135.40, 138.1, 138.2, 141.45, 141.52, 141.98, 142.01, 163.82, 163.84, 167.28, 167.33 ppm (56 signals for 78 different carbons). Elem. Anal. for C_39_H_39_NO_4_S_2_: Calcd. C 72.08, H 6.05, N 2.16; found C 71.99, H 6.06, N 2.15.

Diastereoisomers **8bgD1** and **8bgD2**. (*M/P*)-1,3,7-trimethyl[1,4]benzothiazino[2,3,4-*kl*]phenothiazine-2-yl (S)-N-Boc pipecolate. Following the general procedure from **1b(OH)** (40 mg, 0.11 mmol) and (S)-N-Boc pipecolic acid **7g** (25 mg, 0.11 mmol), kept for 3 h at room temperature. The crude was purified by flash chromatography on silica gel (petroleum ether/CH_2_Cl_2_ 1/3, D1 *Rf* 0.27, D2 *Rf* 0.20) to afford the product **8bgD1** (17 mg, 28% yield) as a white solid (mp 79–82 °C) and the product **8bgD2** (9 mg, 15% yield) as a white solid (mp 121–125 °C). **8bgD1**: ^1^HNMR (400Mz, CDCl_3_) δ: 1.10–1.21 (m, 1H), 1.26–1.53 (m, 14H), 2.21 (s, 3H), 2.28 (s, 3H), 2.29 (s, 3H), 2.29 (s, 3H), 2.80–2.95 (m, 1H), 3.79–3.96 (m, 1H), 4.40–4.47 (m, 1H), 6.78–6.91 (m, 6H), 7.00 (bs, 1H) ppm. ^13^C NMR (100 MHz, CDCl3) δ: 20.6, 20.7, 21.1, 21.4, 24.7, 25.0, 26.0, 28.5, 29.8, 41.2, 42.2, 54.3, 55.3, 77.2, 80.0, 80.1, 118.2, 122.6, 123.2, 125.5, 125.8, 126.3, 127.2, 127.7, 127.9, 128.1, 128.5, 129.5, 130.9, 131.3, 133.8, 134.2, 134.8, 134.9, 135.5, 138.1, 141.9, 142.4, 155.4, 155.7, 169.7, ppm. IR (ATR solid) n: 2973, 2924, 2860, 1764, 1689, 1480, 1448, 1364, 1252 cm^−1^. ${\left[\alpha \right]}_{D}^{20}$ −157, (*c* 0.1, CH_2_Cl_2_). **8bgD2**: ^1^HNMR (400Mz, CDCl_3_) δ: 0.79–0.99 (m, 1H), 1.26–1.45 (m, 14H), 2.14–2.29 (m, 9H), 2.46–2.94 (m, 1H), 3.65–3.92 (m, 1H), 4.39–4.63 (m, 1H), 6.78–6.91 (m, 6H), 7.00–7.02 (m, 1H) ppm. ^13^C NMR (100 MHz, CDCl3) δ: 20.1, 20.2, 20.5, 20.6, 20.70, 20.74, 20.8, 21.0, 24.7, 24.9, 26.0, 26.1, 28.4, 28.6, 41.0, 42.0, 54.4, 55.3, 80.0, 80.3, 115.2, 118.1, 118.2, 122.7, 122.9, 125.5, 125.8, 126.4, 127.5, 128.1, 128.3, 128.9, 129.5, 131.5, 133.8, 134.9, 135.6, 138.1, 142.5, 155.7, 169.7, 169.9, ppm. ${\left[\alpha \right]}_{D}^{20}$ +49 (*c* 0.1, CH_2_Cl_2_).

Diastereoisomers **8bhD1** and **8bhD2**. (*M/P*)-1,3,7-trimethyl[1,4]benzothiazino[2,3,4-*kl*]phenothiazine-2-yl (1*S*)-camphanate. Following the general procedure from **1b(OH)** (259 mg, 0.71 mmol) and (1*S*)-(−)-camphanic acid **7h** (170 mg, 0.86 mmol), kept for 22 h at room temperature. The crude was purified by flash chromatography on silica gel (petroleum ether/CH_2_Cl_2_ 1/3, D1 *Rf* 0.37, D2 *Rf* 0.26) to afford product **8bhD1** (143 mg, 37% yield) as a white solid (mp 70–72 °C) and product **8bhD2** (126 mg, 33% yield) as a white solid (mp 86–88 °C). **8bhD1**: ^1^HNMR (400 MHz, CDCl_3_) δ: 0.97 (s, 3H), 0.98 (s, 3H), 1.06 (s, 3H), 1.23–1.30 (m, 1H), 1.50–1.55 (m, 1H), 1.77–1.84 (m, 1H), 1.98–2.05 (m, 1H), 2.22 (s, 3H), 2.26 (s, 3H), 2.32 (s, 3H), 6.73 (bs, 1H), 6.79–6.81 (m, 2H), 6.85 (bs, 1H), 6.91–6.96 (m, 2H), 7.00 (bs, 1H) ppm. ^13^C NMR (100 MHz, CDCl_3_) δ: 9.8, 16.9, 17.0, 20.6, 20.7, 20.8, 29.0, 29.7, 54.5, 54.8, 90.8, 118.2, 122.7, 125.46, 125.49, 126.0, 126.2, 126.5, 127.1, 127.9, 128.7, 129.8, 131.6, 134.5, 135.0, 135.7, 137.9, 141.3, 141.6, 164.5, 177.9. IR (ATR solid) n: 2970, 2922, 2867, 1787, 1776, 1481, 1449, 1309, 1250 cm^−1^. ${\left[\alpha \right]}_{D}^{20}$ −129 (*c* 0.1, CH_2_Cl_2_). **8bhD2** ^1^HNMR (400 MHz, CDCl_3_) δ: 0.89 (s, 3H), 0.93 (s, 3H), 1.06 (s, 3H), 1.51–1.56 (m, 3H), 1.65–1.71 (m, 1H), 2.21 (s, 3H), 2.25 (s, 3H), 2.31 (s, 3H), 6.68 (bs, 1H), 6.77–6.79 (m, 2H), 6.84–6.89 (m, 2H), 6.96 (bs, 1H), 7.02 (bs, 1H) ppm. ^13^C NMR (100 MHz, CDCl_3_) δ: 9.8, 16.9, 17.0, 20.6, 20.7, 20.8, 28.9 29.6, 54.3, 54.8, 90.8, 118.1, 122.7, 125.2, 125.9, 126.3, 126.6 (2C), 127.5, 128.0, 128.2, 129.9, 132.0, 133.9, 135.0, 135.7, 137.8, 141.6, 142.1, 164.5, 177.7 ppm. IR (ATR solid) n: 2967, 2922, 2865, 1776, 1482, 1449, 1309, 1250 cm^−1^. ${\left[\alpha \right]}_{D}^{20}$ + 126 (*c* 0.1, CH_2_Cl_2_).

General Procedure for the hydrolysis: To a solution of ester **8** in CH_2_Cl_2_/MeOH: 10/1 (roughly 0.05 M) 3 eq. of NaOH was added, and the solution was stirred for 4–6 h at room temperature. The solution was diluted with water and HCl (1M) was added until the pH was neutral, then the mixture was extracted with CH_2_Cl_2_ (3 × 5 mL). The organic layer was dried over Na_2_SO_4_, filtered, and then evaporated under reduced pressure. The crude was purified by flash chromatography on silica gel (Petroleum Ether/CH_2_Cl_2_ 1/2) to afford the products (*M*)-**1b(OH)** or (*P*)-**1b(OH)** as a white solid in quantitative yield. (*M*)-**1b(OH)** ${\left[\alpha \right]}_{D}^{20}$ −161 (*c* 0.1, CH_2_Cl_2_) and (*P*)-**1b(OH)** ${\left[\alpha \right]}_{D}^{20}$ +166 (*c* 0.1, CH_2_Cl_2_).

Experimental HPLC Analytical (250 × 4.6 mm) column packed with Chiralpak IA chiral stationary phase was purchased from Chiral Technologies Europe. The HPLC resolution of products was performed on a HPLC Waters Alliance 2695 equipped with a 200 μL loop injector and a spectrophotometer UV Waters PDA 2996. The mobile phase, delivered at a flow rate of 1.2 mL/min, was hexane/CH_2_Cl_2_ 70/30 *v*/*v* + 1% MeOH.

## 4. Conclusions

In this paper we have reported that the fine matching of the structures of the chiral auxiliaries used and, above all, the topology of their insertion on the helical skeleton, *bay-zone* vs *cape-zone*, allow for the chemical resolution of DTA[4]H) **1**. Helicene **1b(OH)** allowed for the insertion of the chiral auxiliary on the 1-position, the area of terminal ring superimposition that we indicated as the *bay-zone*. Esterification of **1b(OH)** with (1*S*)-(−)-camphanic acid **7h** provided diastereomers **8bhD1** and **8bhD2** which were successfully separated by flash chromatography and hydrolyzed providing enantiopure helicenes (*M*)-**1b(OH)** and (*P*)-**1b(OH)**, respectively.

## Figures and Tables

**Table 1:** Diastereomeric esters **8** obtained reaction racemic phenols **1a(OH)** or **1b(OH)** with enantiopure acids **7a**–**h**.

Chiral Auxiliary	Product	Yield	Resolution
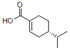 **7a** (*S*)-(−)-Perillic acid	**8aaD1/8aaD2**	60%	No resolution
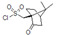 **7b** Camphorsulfonyl chloride	**8abD1/8abD2**	56%	No resolution
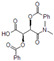 **7c** (−)-o,o’-Dibenzoyl-L-tartaric acid mono(dimethyl amide)	**8acD1/8acD2**	56%	No resolution
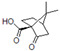 **7d** (1*S*)-(+)-Ketopinic acid	**8adD1/8adD2** **8bdD1/8bdD2**	79% 55%	No resolution No resolution
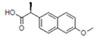 **7e** (*S*)-(+)-2-(6-Methoxy-2-naphthyl)propionic acid	**8aeD1/8aeD2** **8beD1/8beD2**	94% 58%	No resolution No resolution
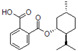 **7f** (-)mono-(1R)-Menthylphthalate	**8afD1/8afD2** **8bfD1/8bfD2**	54% 45%	No resolution No resolution
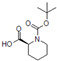 **7g** (S)-N-Boc pipecolic acid	**8agD1/8agD2** **8bgD1/8bgD2**	69% 28%15%	No resolution Chromatographic resolution is possible
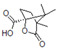 **7h** (1*S*)-(−)-Camphanic acid	**8ahD1/8ahD2** **8bhD1/8bhD2**	57% 37% 33%	No resolution Chromatographic resolution is possible

## Data Availability

Not applicable.

## Supplementary Materials

The following supporting information is available online: HPLC Analysis, NMR spectra, DFT calculations of compound **1b(OH)**, Optimized structures’ coordinates.

## Sample Availability

Samples of the compounds are available from the authors.
