# Supplementary material for: Resolution of a Configurationally Stable Hetero[4]helicene
Source: Molecules. 2022 Feb 9;27(4):1160. doi: 10.3390/molecules27041160 (PMC8874595; doi:10.3390/molecules27041160)
Supplement: Supplementary file 1 [file molecules-27-01160-s001.zip › molecules-1558652-supplementary.pdf]

Resolution of a Configurationally Stable Hetero[4]helicene

Michela Lupi [1], Martina Onori [1], Stefano Menichetti [1], Sergio Abbate [2], Giovanna Longhi [2] and Caterina Viglianisi [1,*]

[1] Department of Chemistry "Ugo Schiff" (DICUS), University of Florence, Via della Lastruccia 13, Sesto Fiorentino (FI), 50019 Florence, Italy; michela.lupi@unifi.it (M.L.); martinaonori@gmail.com (M.O.); stefano.menichetti@unifi.it (S.M.)

[2] Department of Molecular and Translational Medicine (DMMT), University of Brescia, V.le Europa 11 Brescia (BS), 25121 Brescia, Italy; sergio.abbate@unibs.it (S.A.); giovanna.longhi@unibs.it (G.L.)

* Correspondence: caterina.viglianisi@unifi.it

HPLC Analysis

The HPLC resolution of products was performed on a HPLC Waters Alliance 2695 equipped with a 200 µL loop injector and a spectrophotometer UV Waters PDA 2996.

CHIRALPAK® IA (250 x 4.6 mm/ 5µm) purchased from Chiral Technologies Europe.

The mobile phase, delivered at a flow rate 1.2 mL/min, was hexane/CH_2Cl_2 70/30 v/v + 1% MeOH.

Racemate

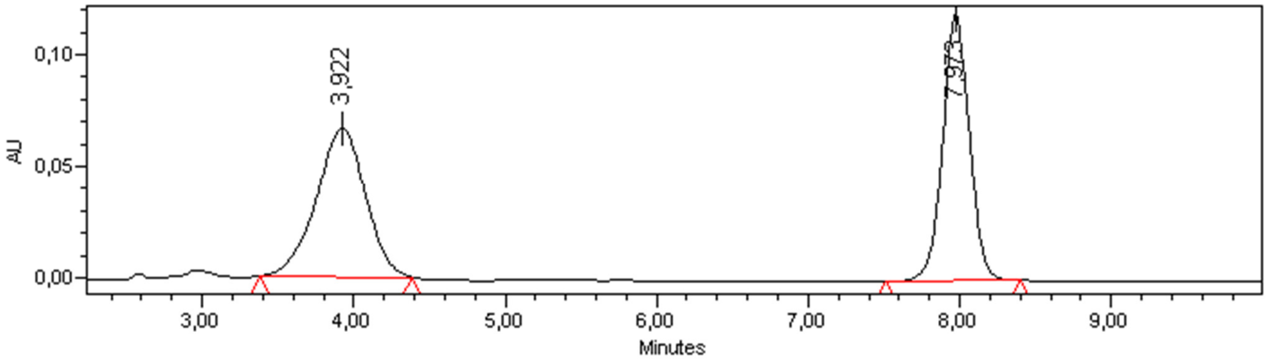

1b(OH) Racemic	Retention time (min)	%Area
(+)	3.922	50.88
(-)	7.973	49.12

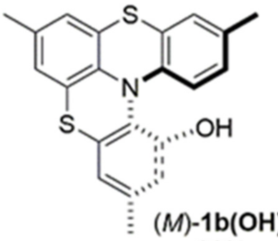

(*M*)-**1b(OH)**
ee>99%

$[\alpha]_D^{20}$ -167<br><br>(*c* 0.1, CH_2Cl_2)	Retention time (min)	%Area
**(*M*)-1b**(OH) (−)	8.001	100.00

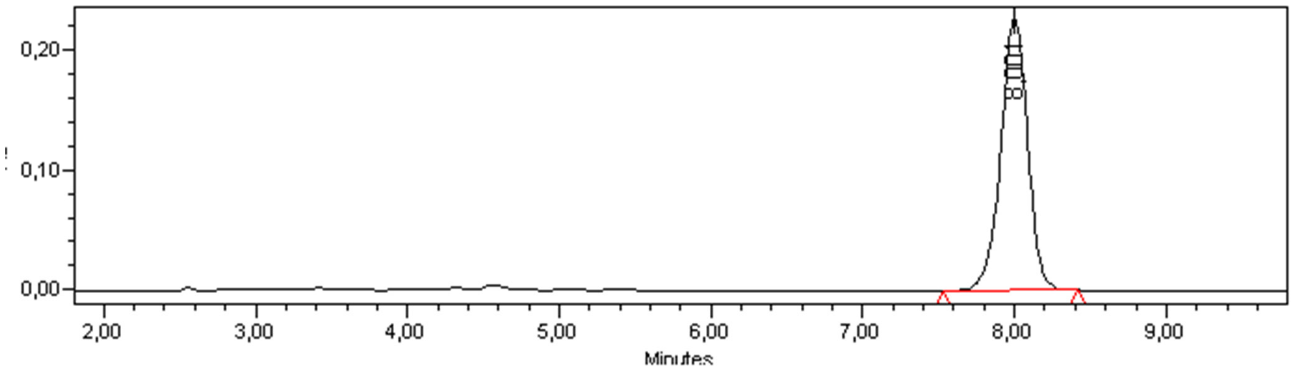

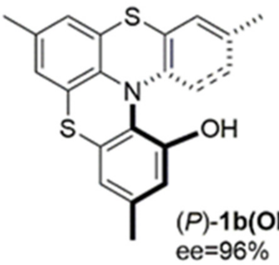

(*P*)-**1b(OH)**
ee=96%

$[\alpha]_D^{20}$ +166<br><br>(*c* 0.1, CH_2Cl_2)	Retention time (min)	%Area
(*P*)-**1b**(OH) (+)	3.799	97.80
(*M*)-**1b**(OH) (−)	8.611	2.20

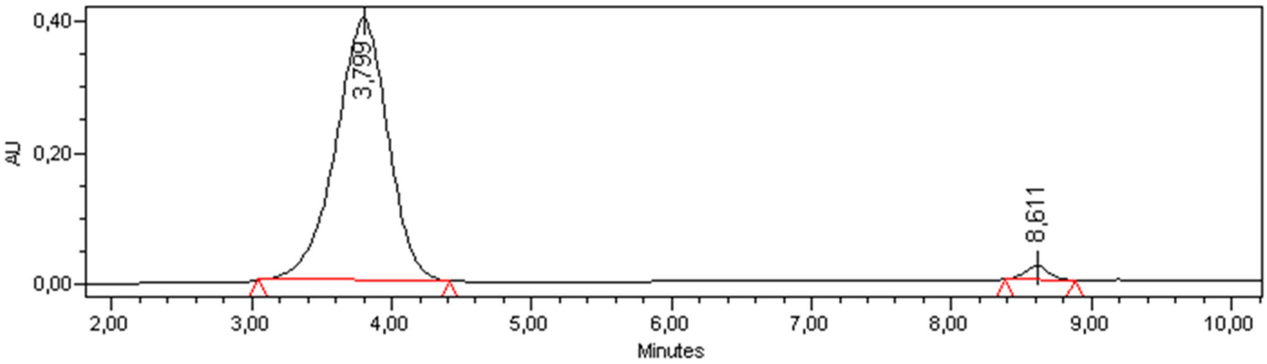

NMR spectra 400 MHz

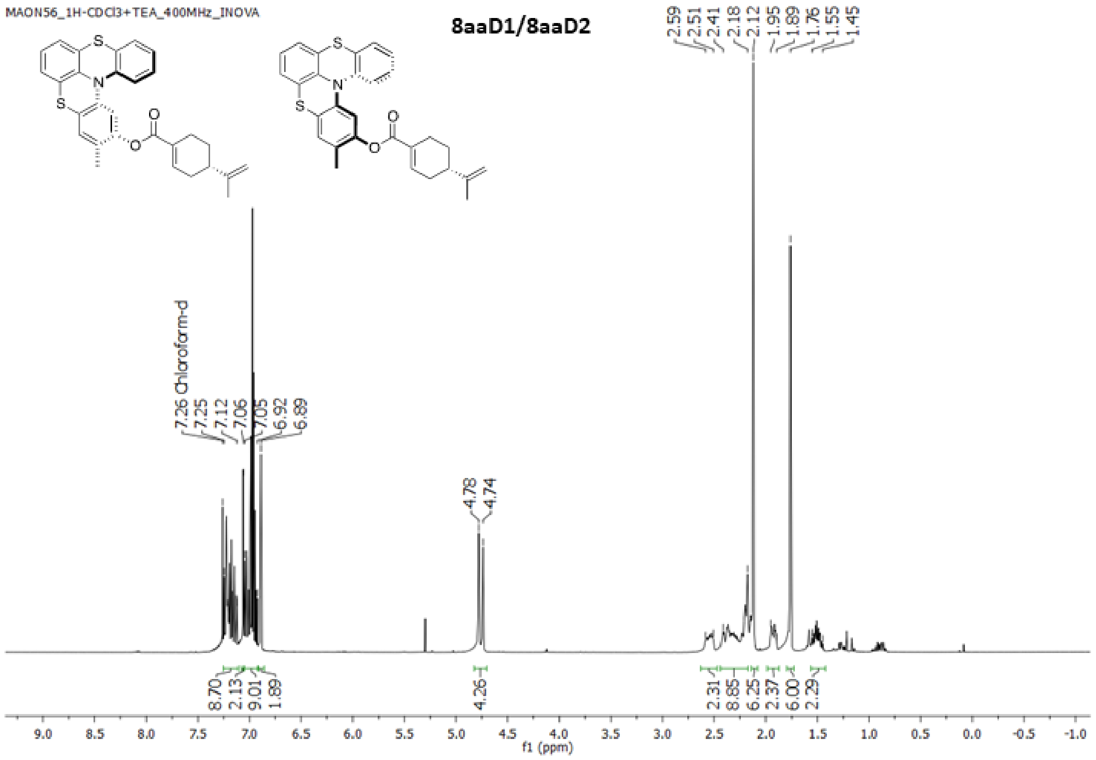

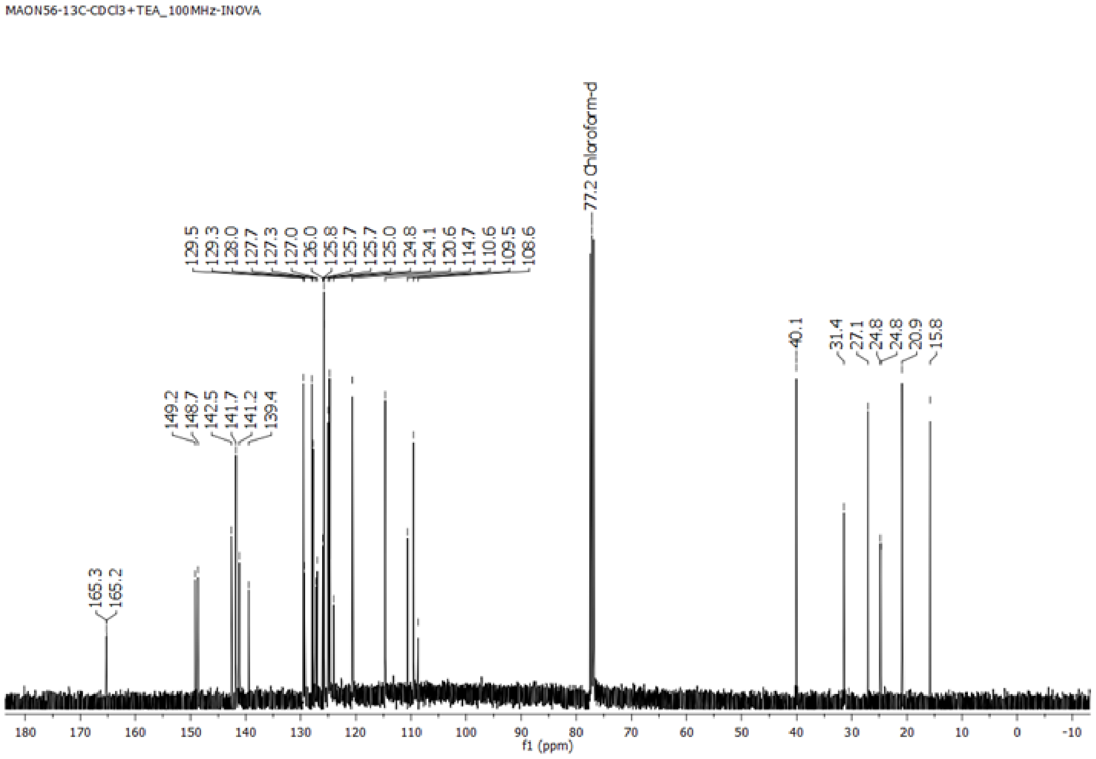

MAON53-f2-1H-400MHz-CDCl3

8abD1/8abD2

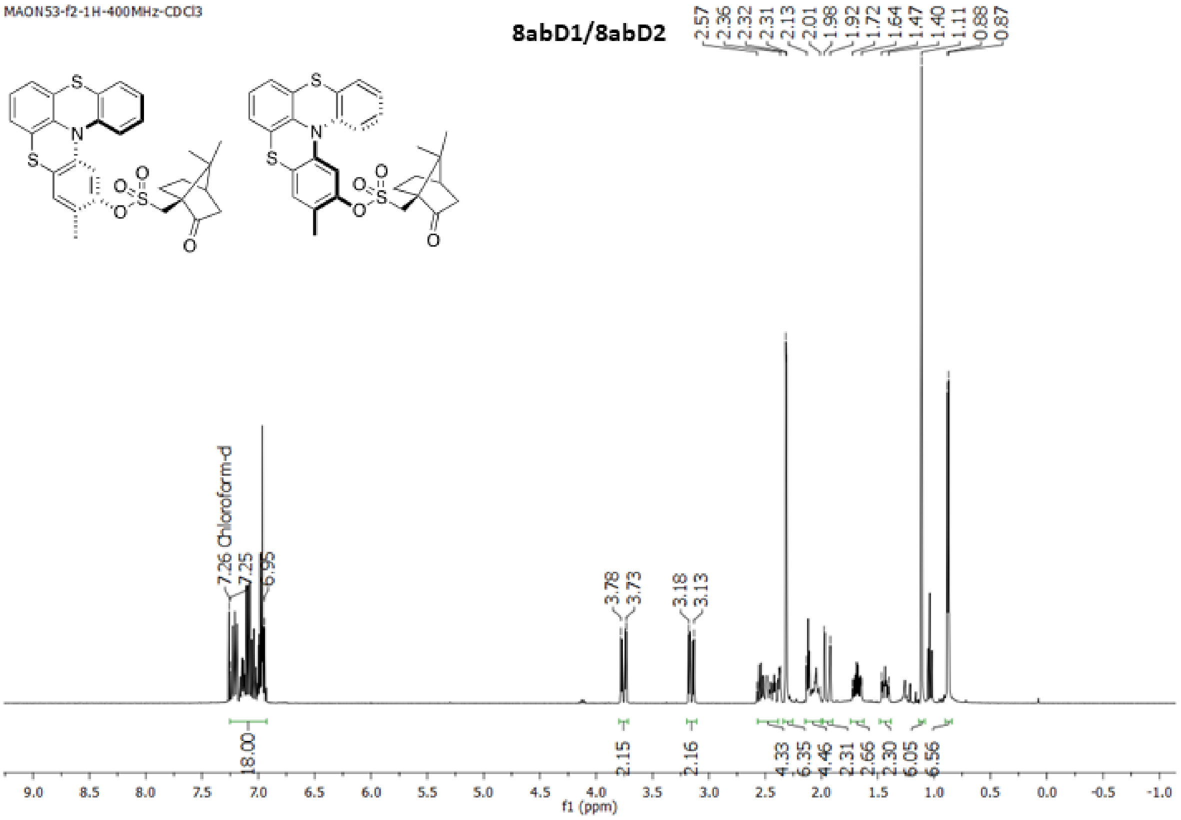

MAON53_f2_28-48_CDCl3_TEA_13C_400MHz

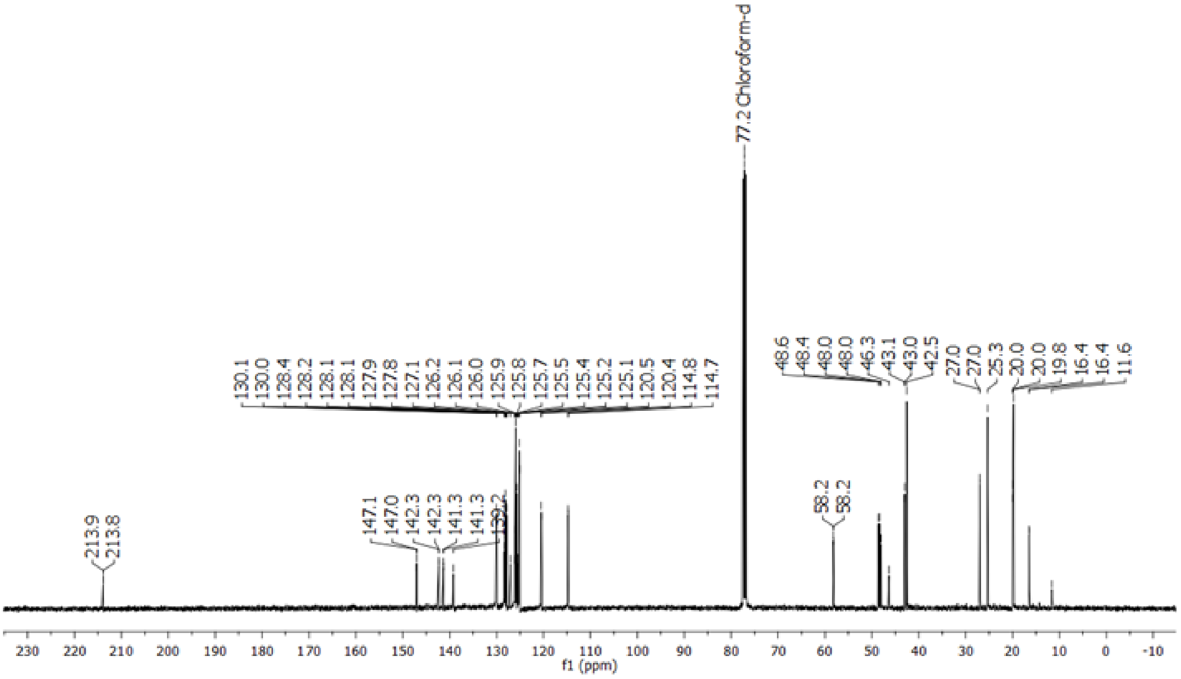

LM215_F2_CDCl3_1HNMR_400MHz

8acD1/8acD2

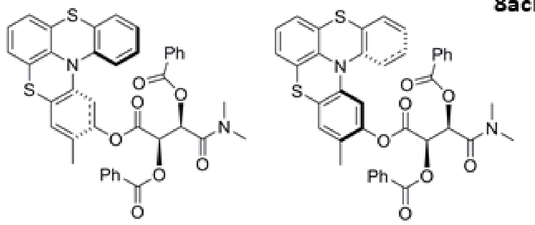

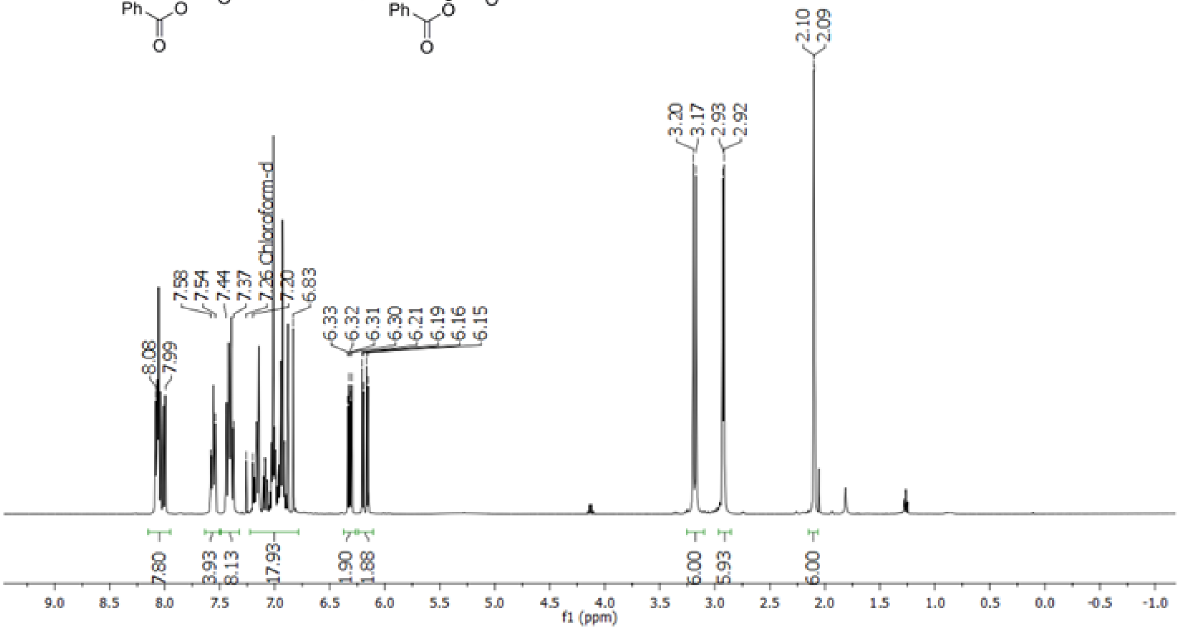

LM215_F2_CDCl3_13CNMR_100MHz

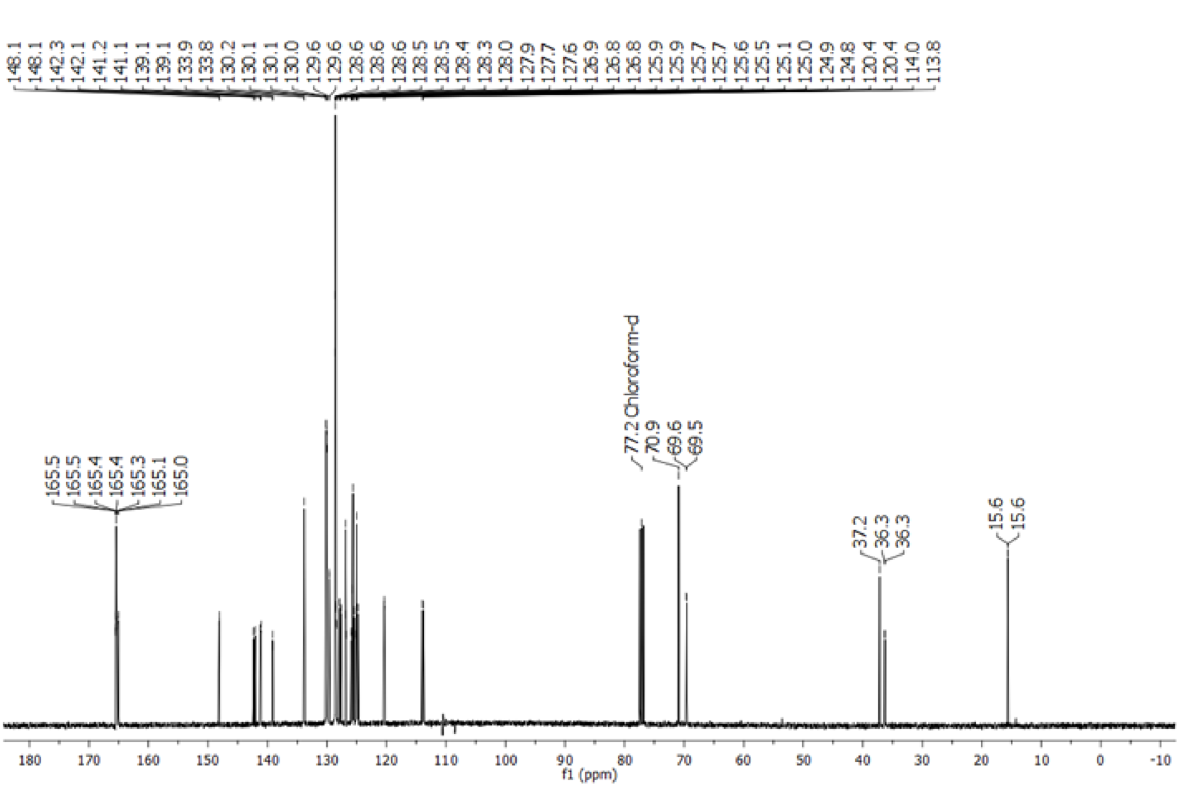

MAON68-f1_1H-CDCl3+TEA_400MHz-INOVA

8adD1/8adD2

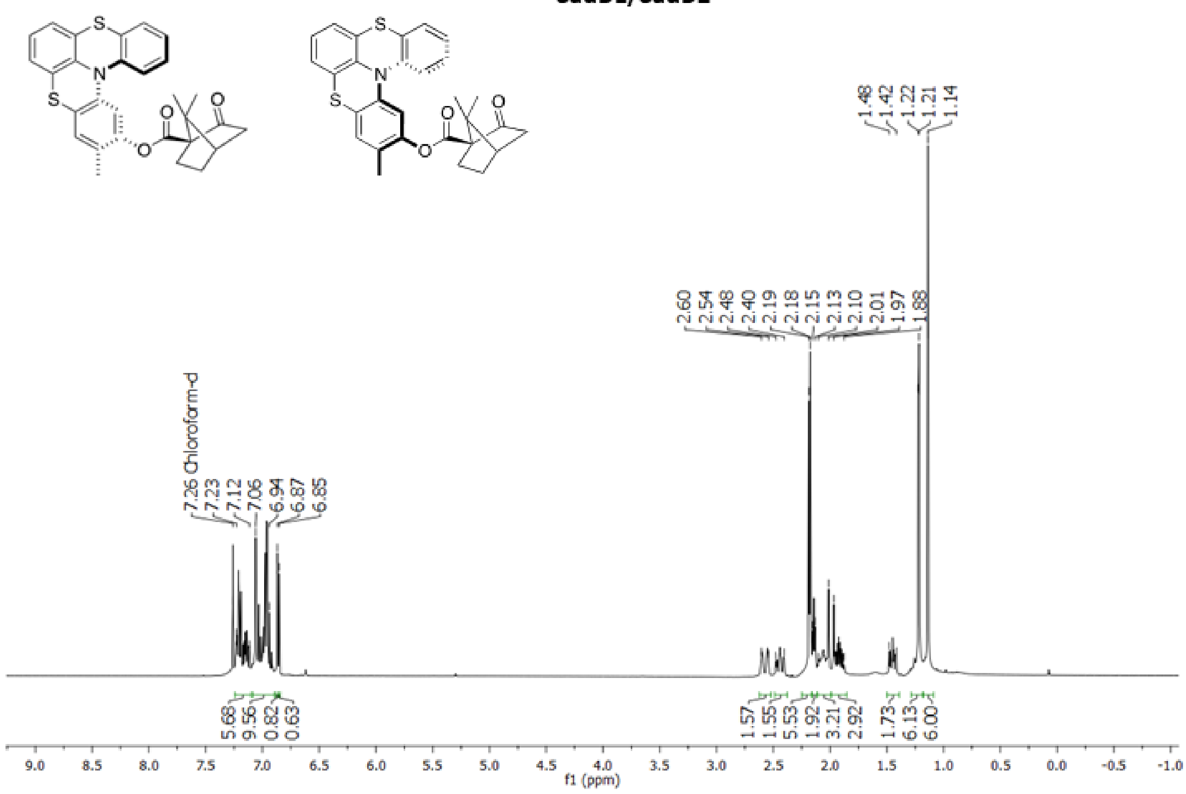

MAON68-f1_13C_CDCL3+TEA_100MHz-INOVA

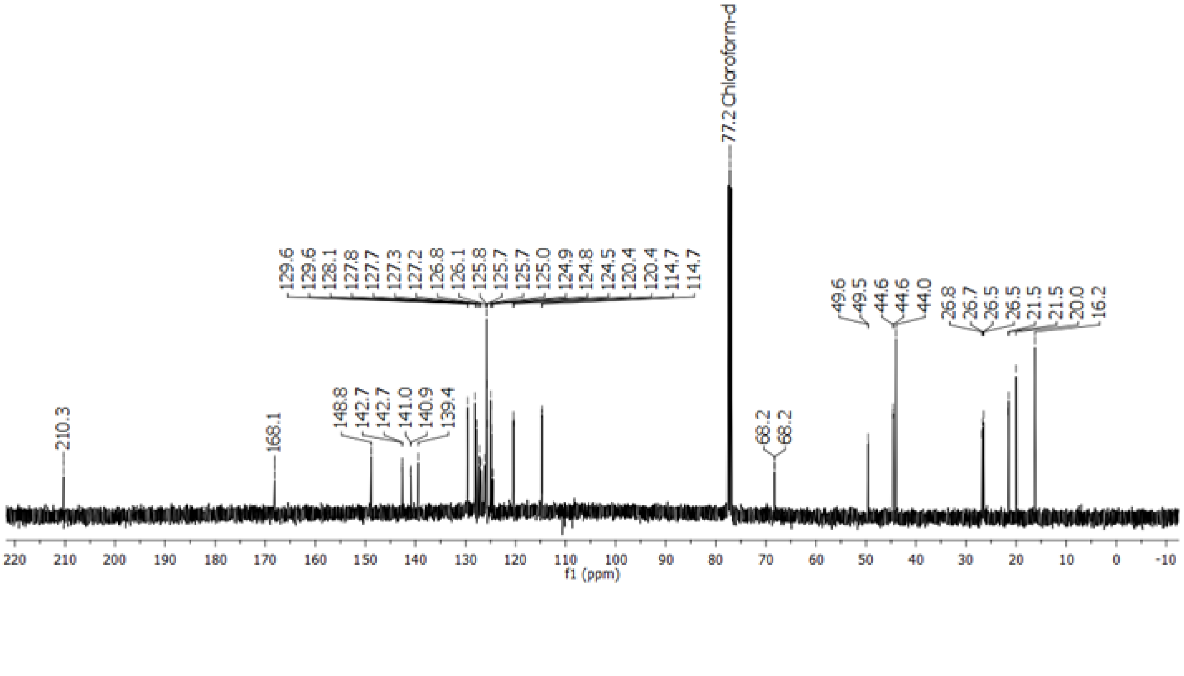

MAON8_CDCl3_TEA_400MHz-INOVA

8aeD1/8aeD2

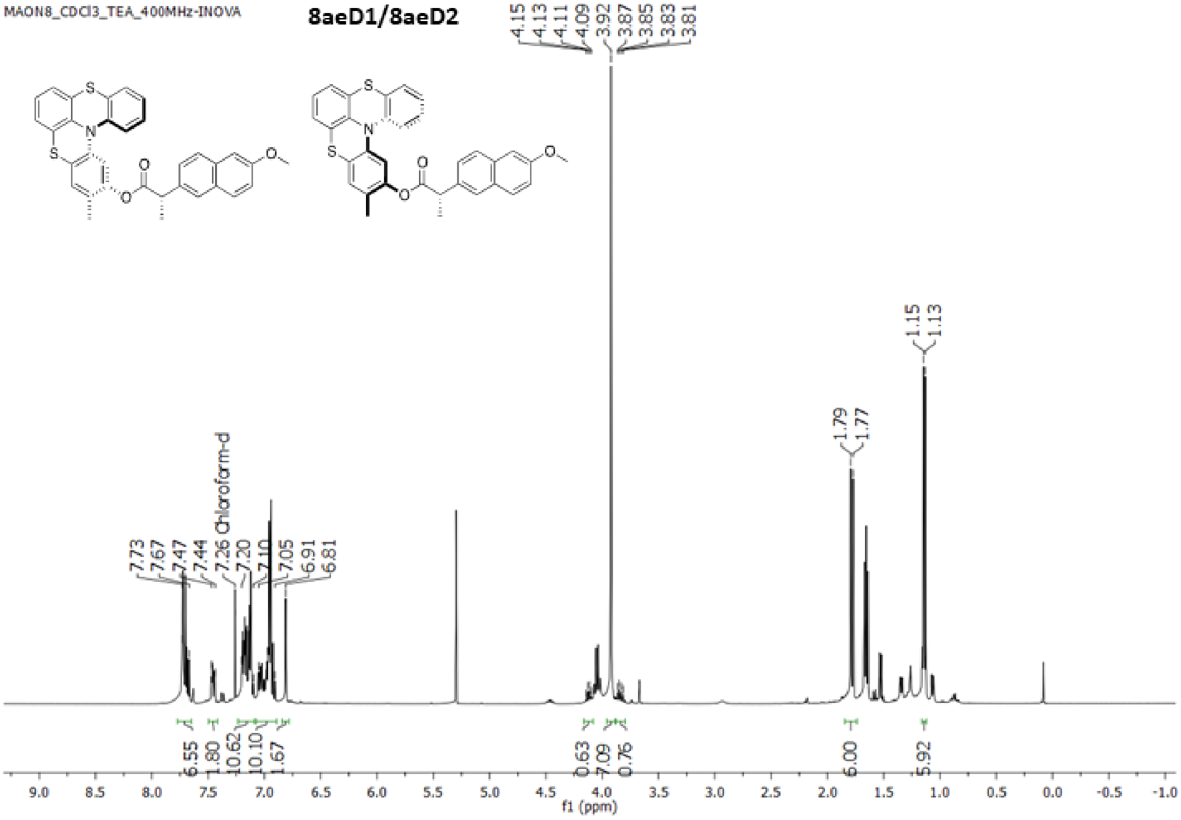

MAON8-CDCl3+TEA_100MHz-INOVA

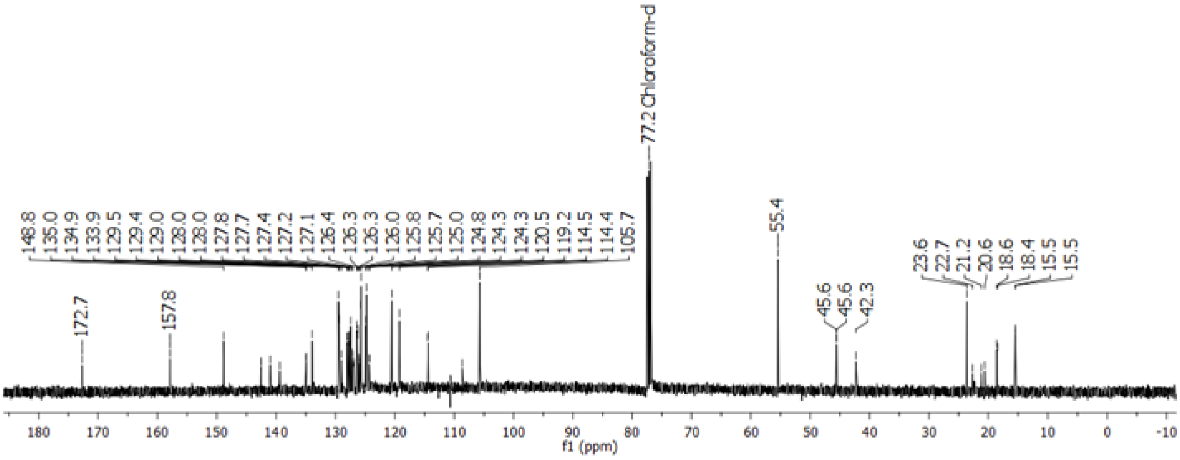

LM219-2col-F3-CDCl3-1HNMR-400MHz

8afD1/8afD2

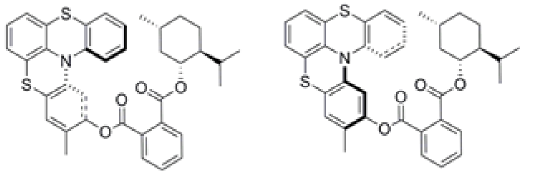

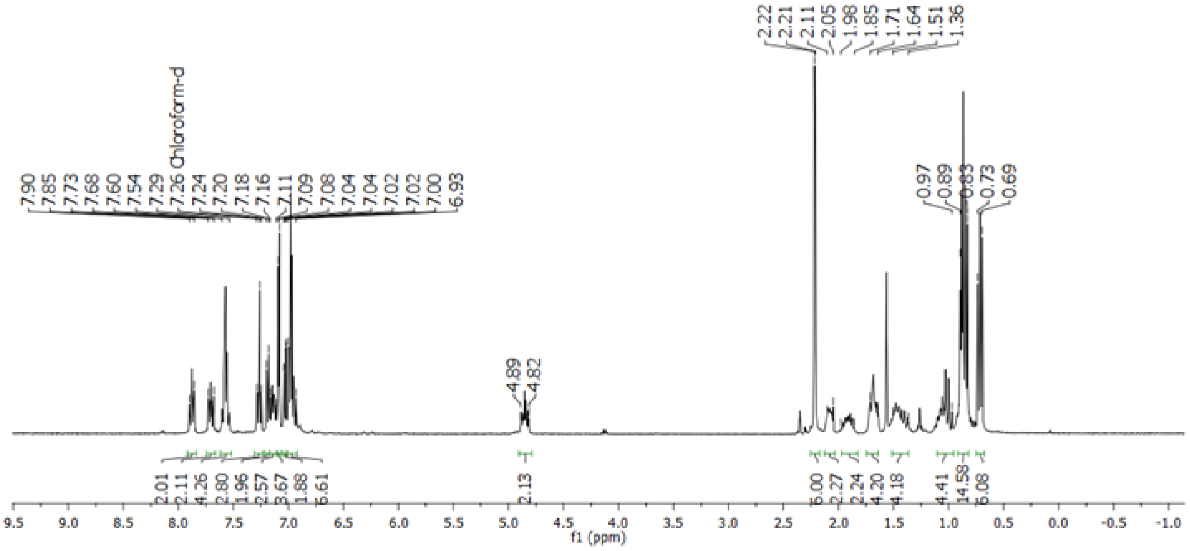

LM219-2col-F3-CDCl3-13CNMR-100MHz

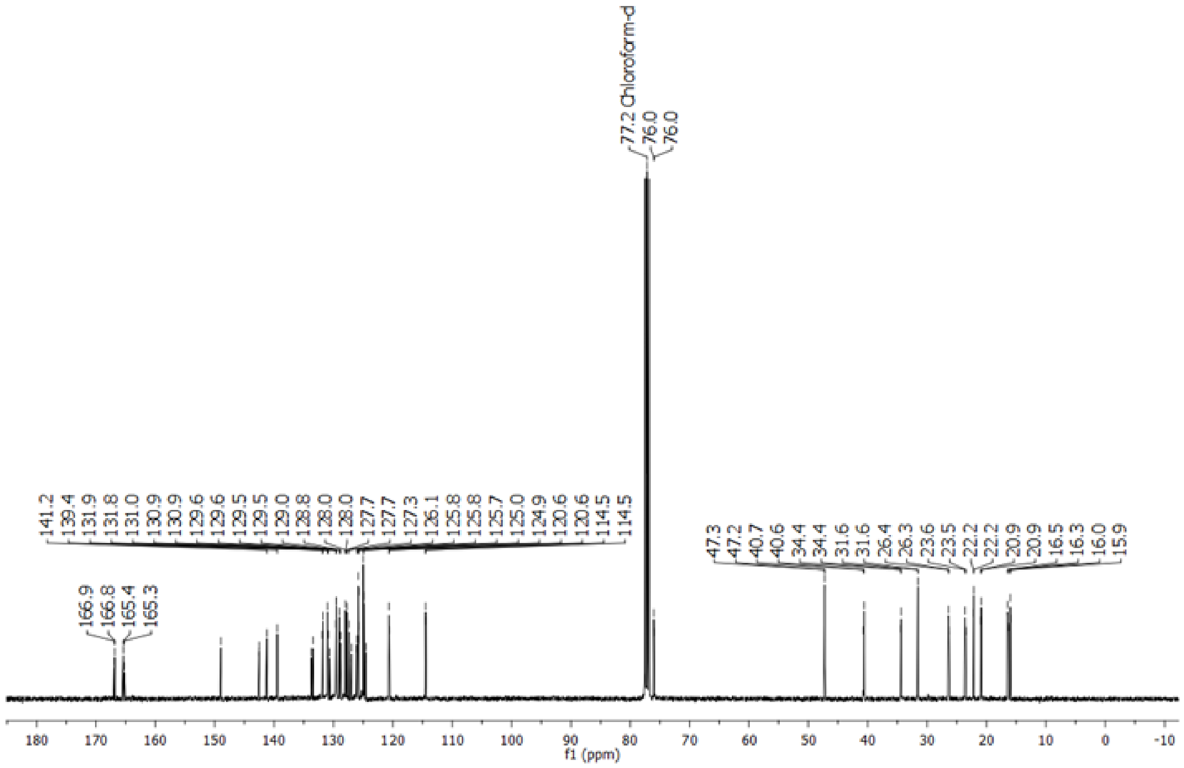

MAON11_1H_400MHz_CDCl3-TEA_14-04-2014

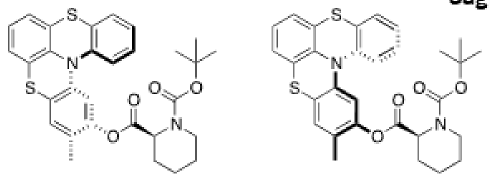

8agD1/8agD2

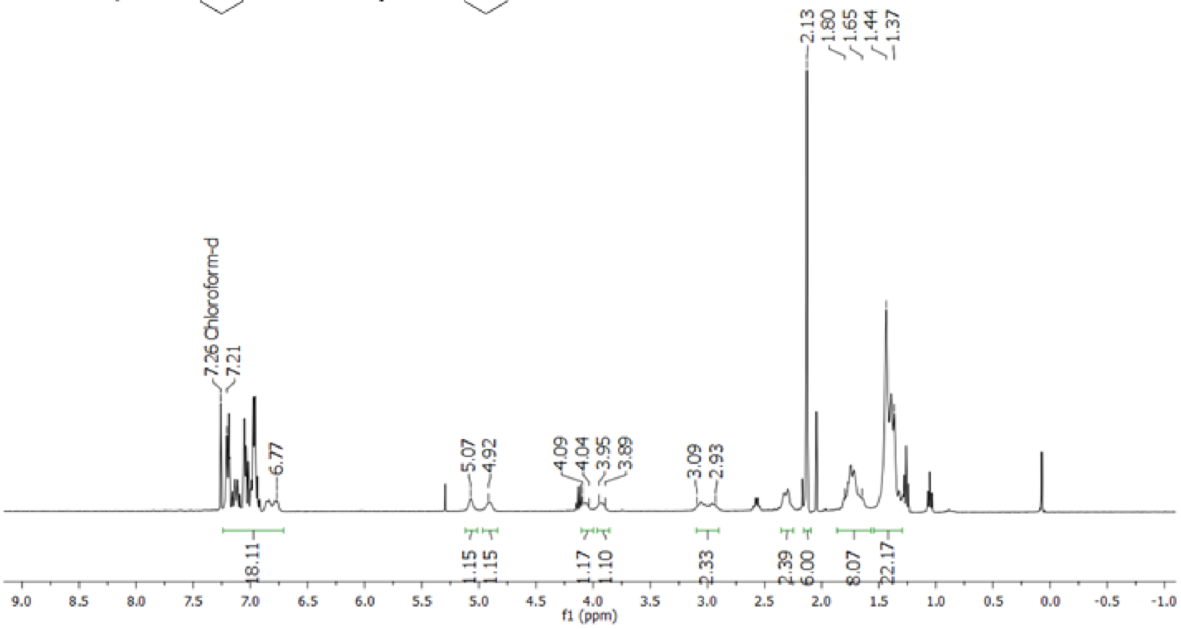

MAON87_f1_1H_400MHz_CDCl3+TEA

8ahD1/8ahD2

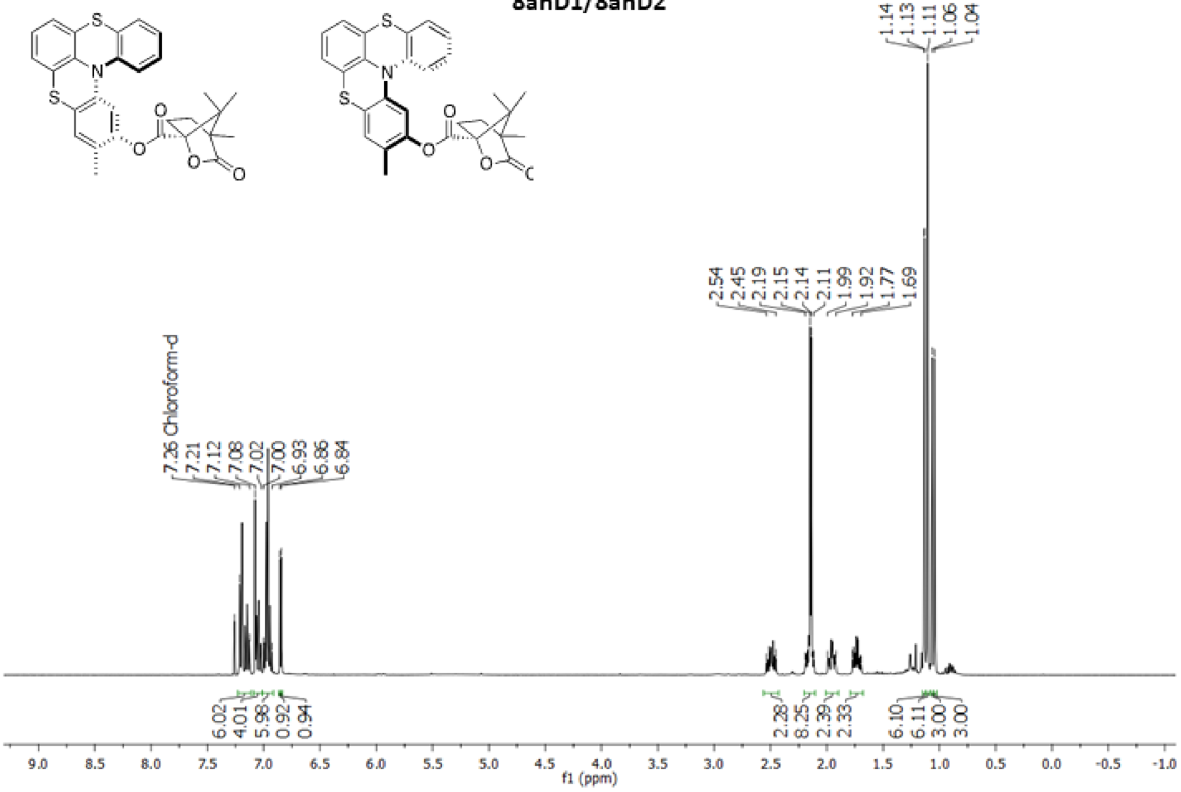

MAON87-f1-13C-100MHz-CDCl3

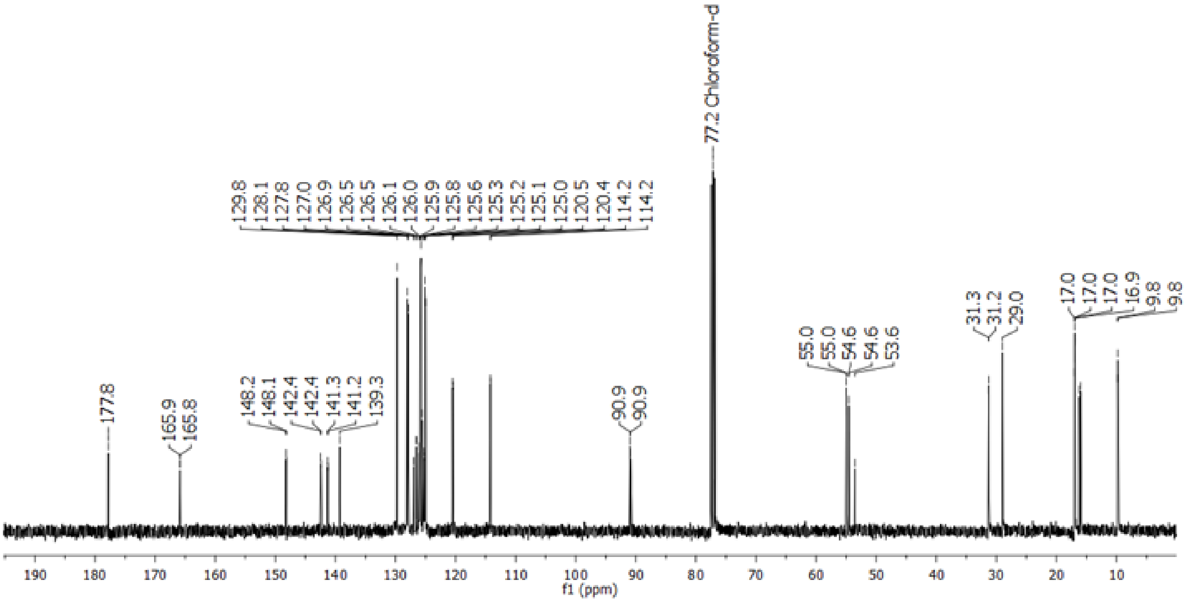

LM243ketopinico-F2-CDCl3-1HNMR-400MHz

8bdD1/8bdD2

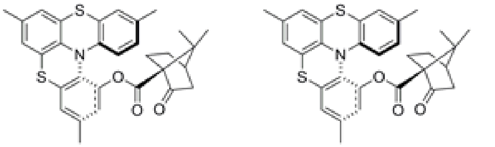

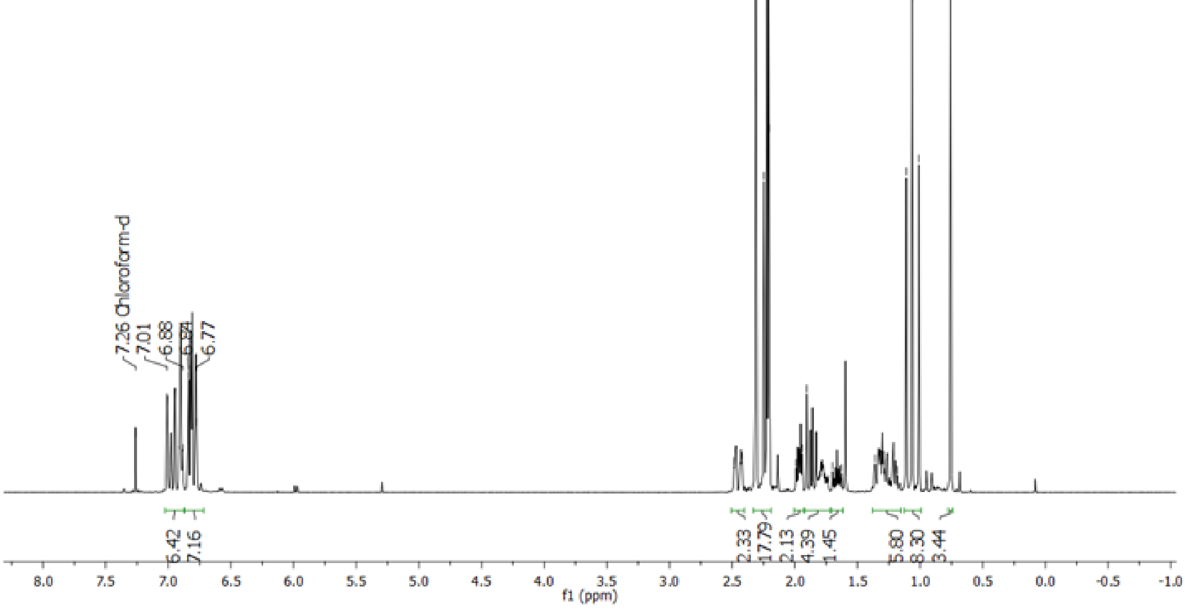

LM252-ketopinico-F2-CDCl3-13CNMR-100MHz

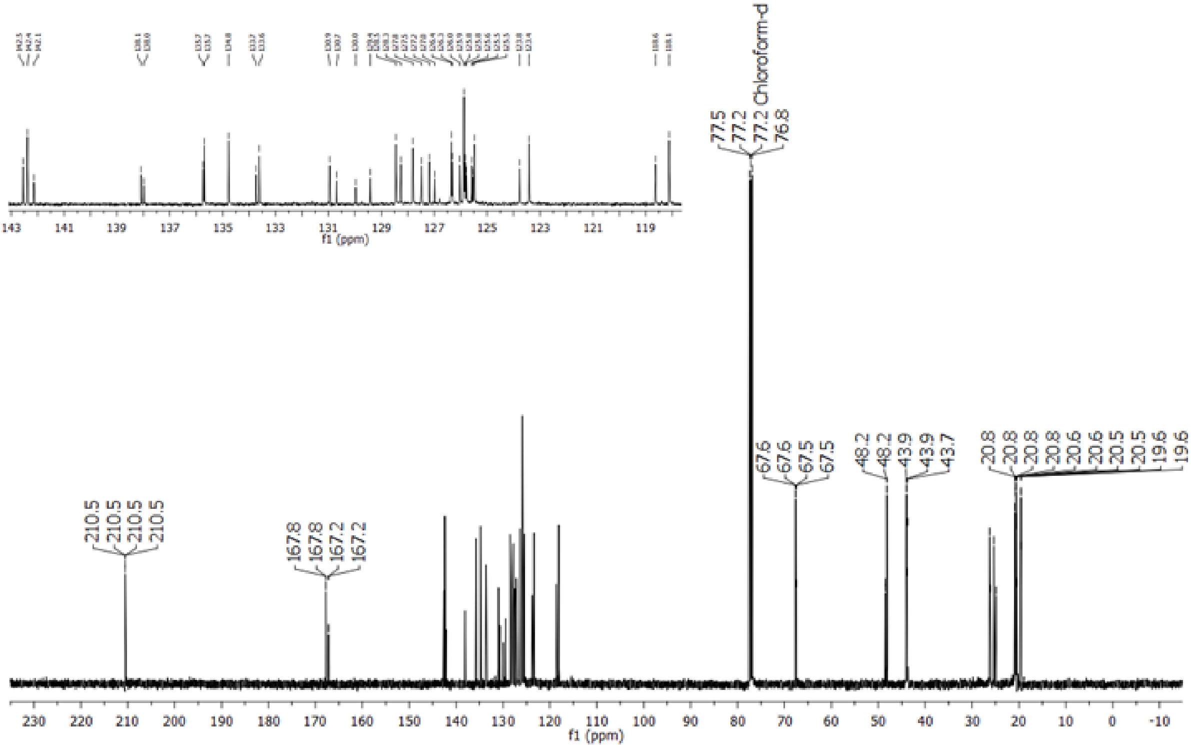

LM245_CDCl3_1HNMR_400MHz

8beD1/8beD2

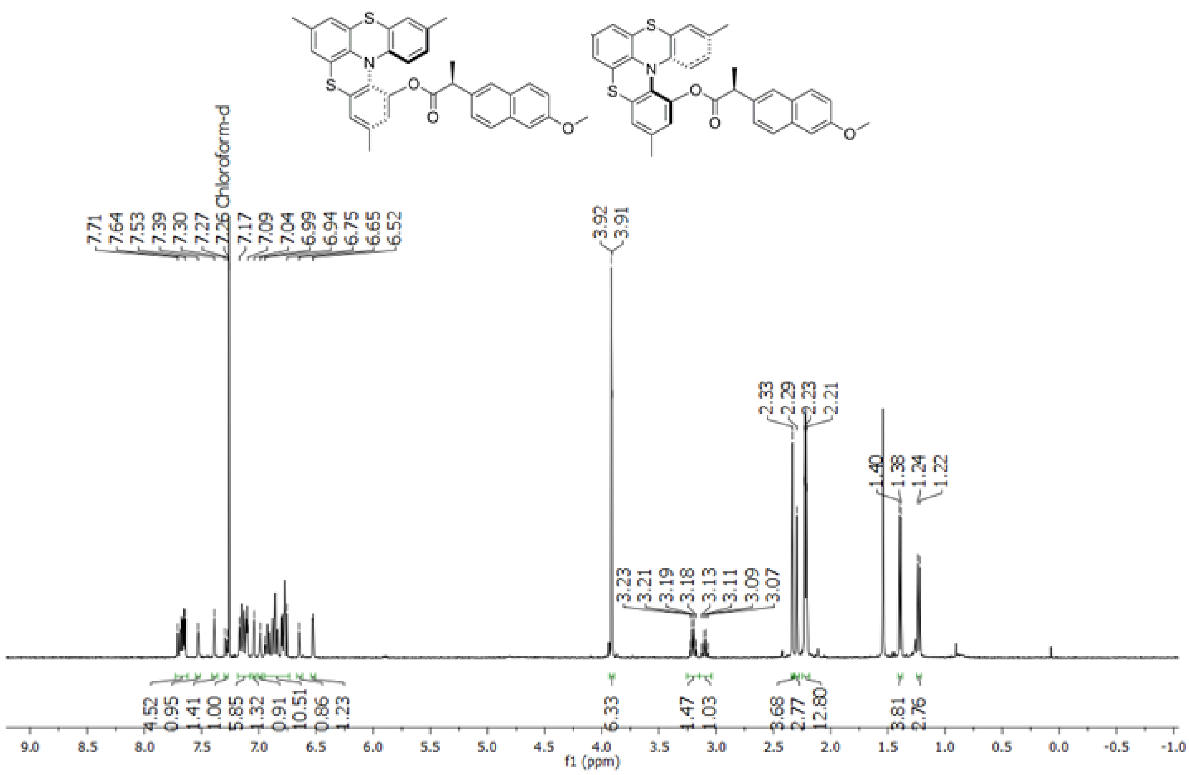

LM244-F3-1HNMR-CDCl3-400MHz

8bfD1/8bfD2

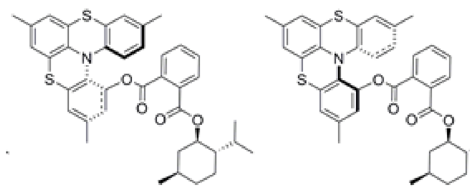

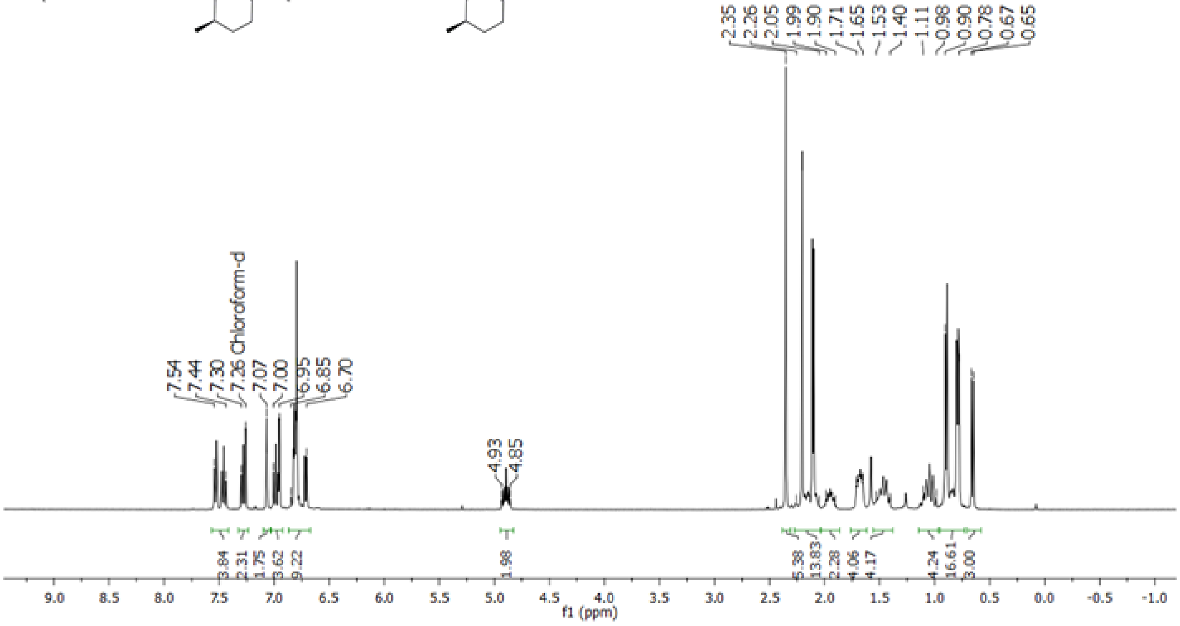

LM244-F3-13CNMR-CDCl3-100MHz

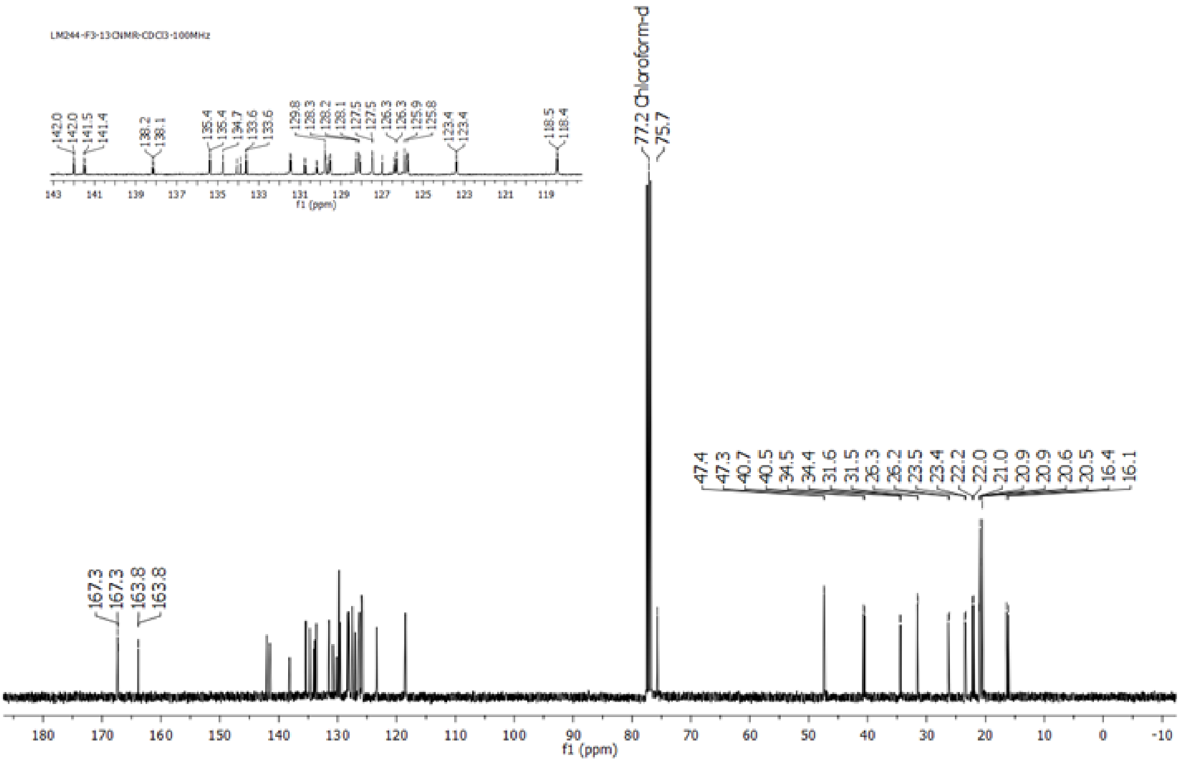

LM242-F1-CDCl3-1HNMR-400MHz

(-)-8bgD1

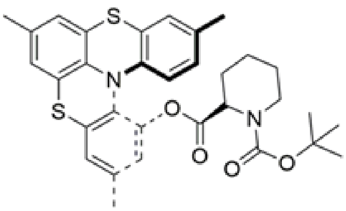

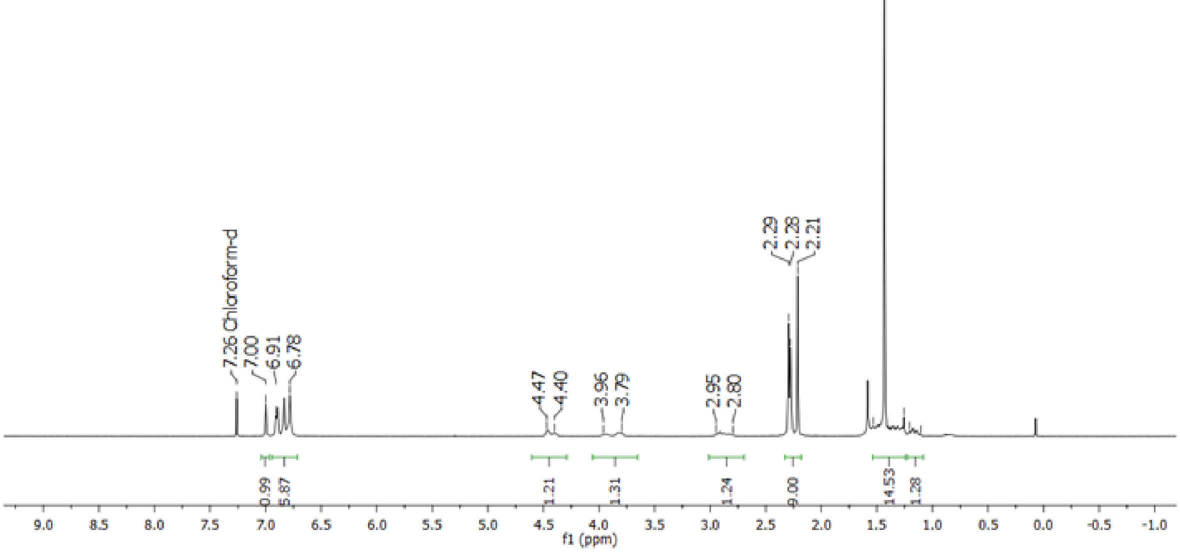

LM242-F1-CDCl3-13CNMR-100MHz-weekend

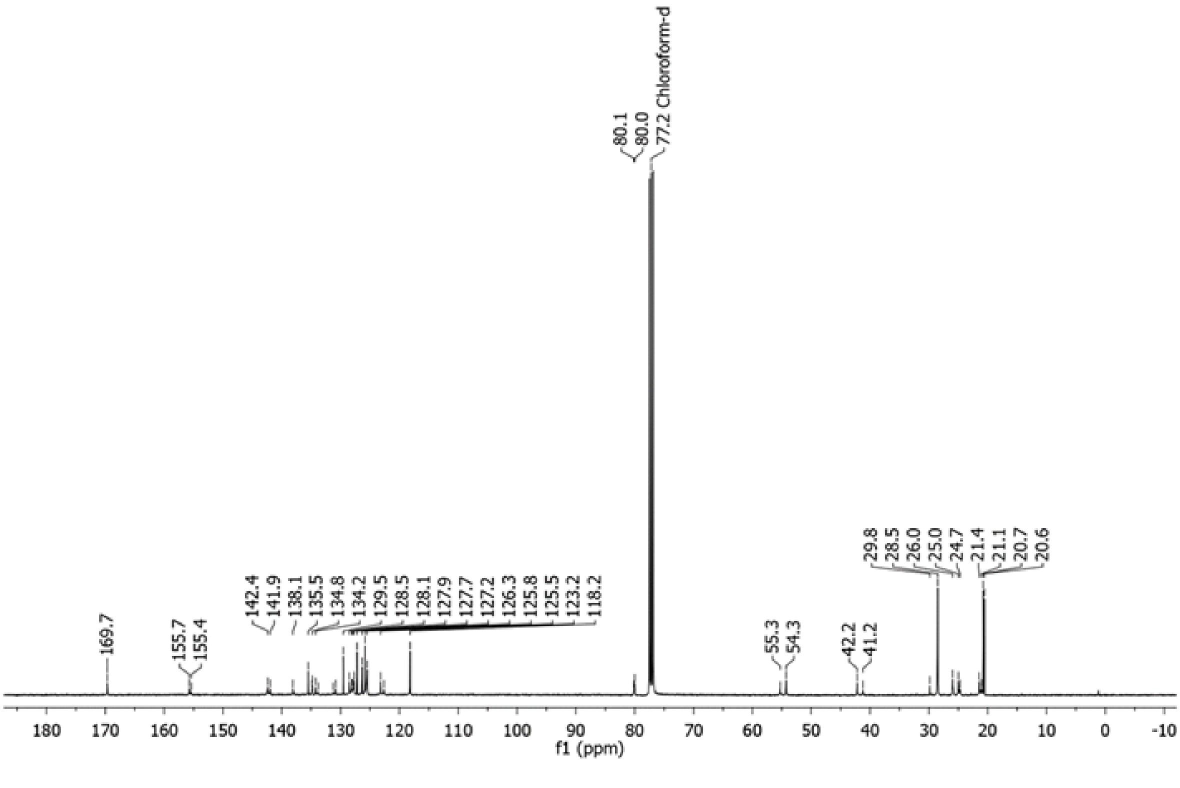

LM242-F2-CDCl3-1HNMR-400MHz

(+)-8bgD2

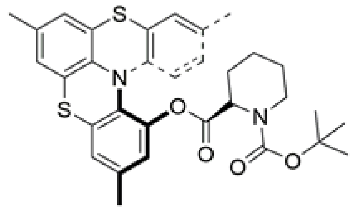

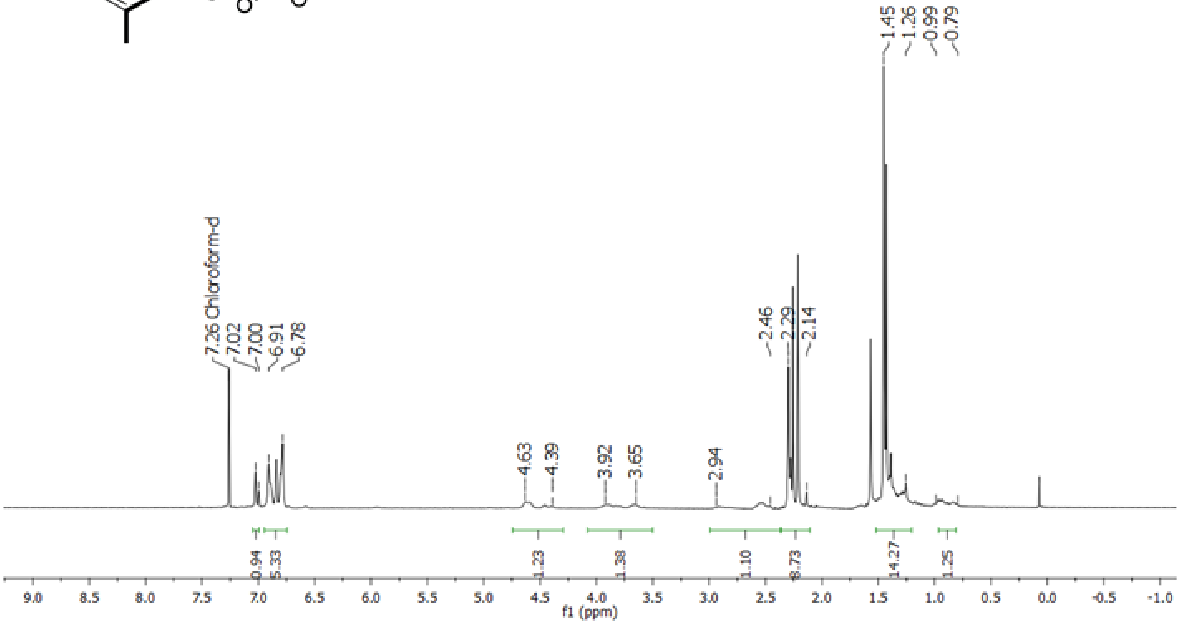

LM325_F3_CDCl3_13CNMR_100MHz

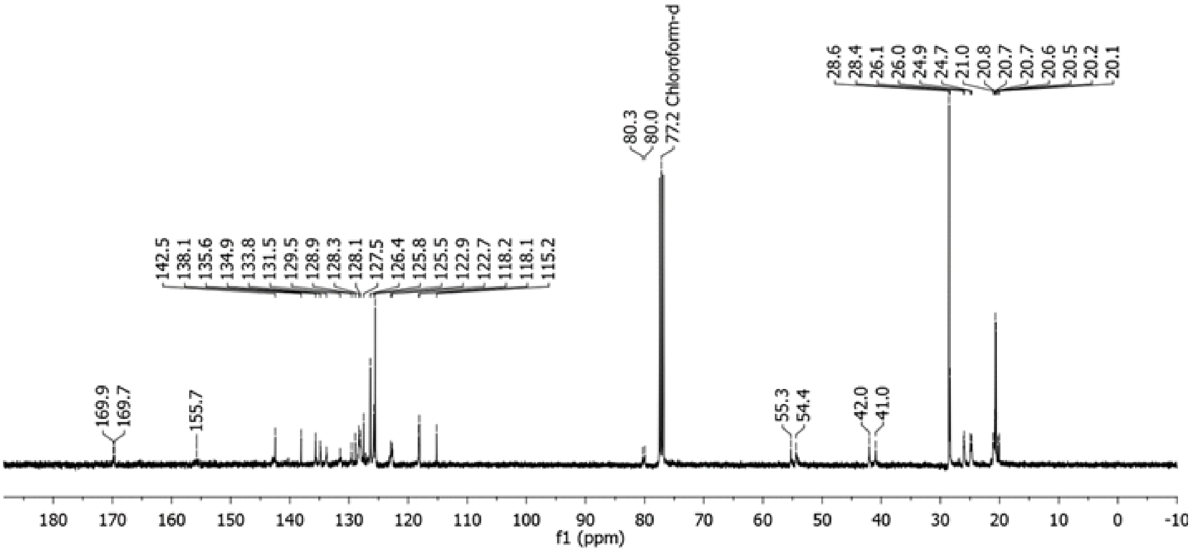

LM251-F3-CDCl3-1HNMR-400MHz

(-)-8bhD1

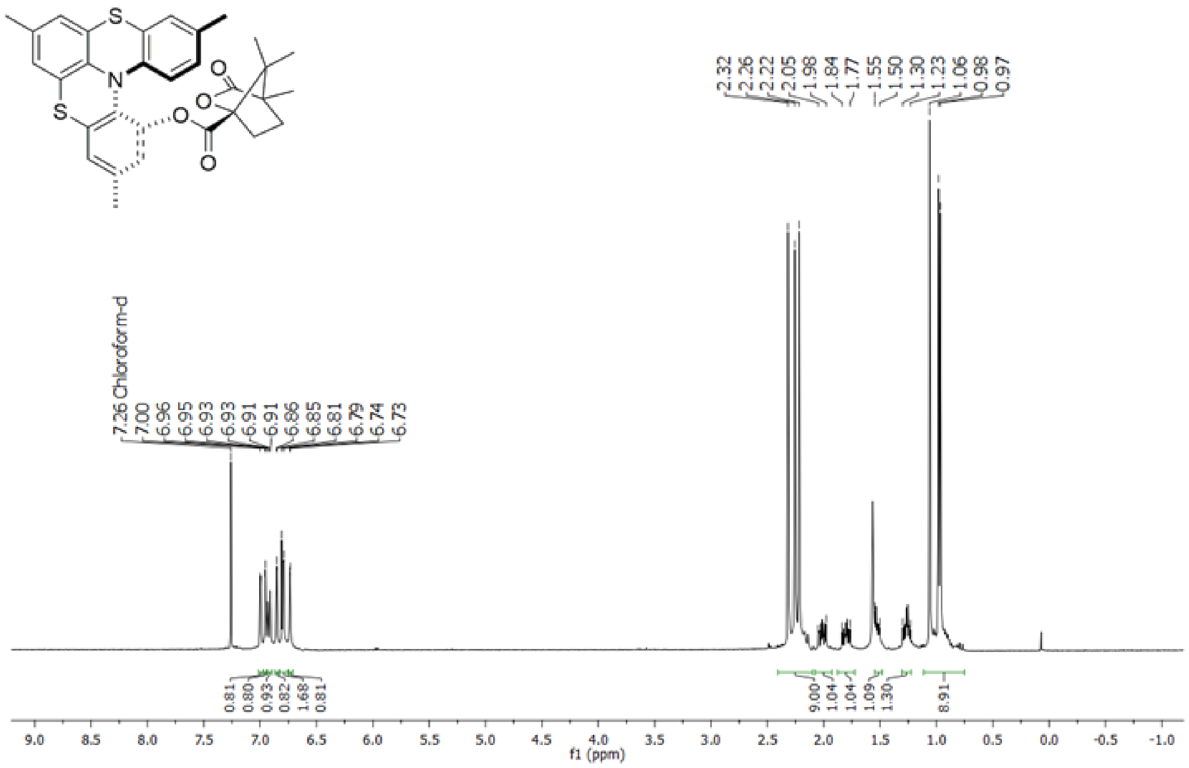

LM251-F3-CDCl3-13CNMR-100MHz

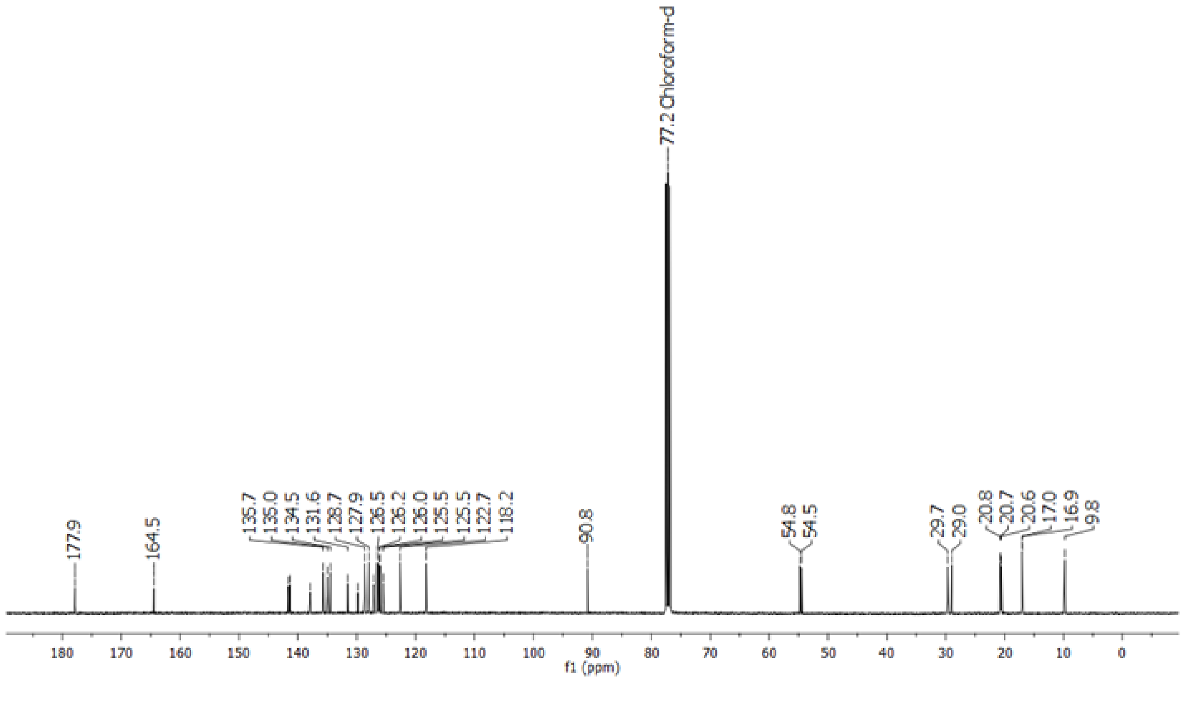

LM251-2diastero-F5-CDCl3-1HNMR-400MHz

(+)-8ahD2

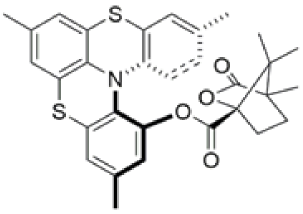

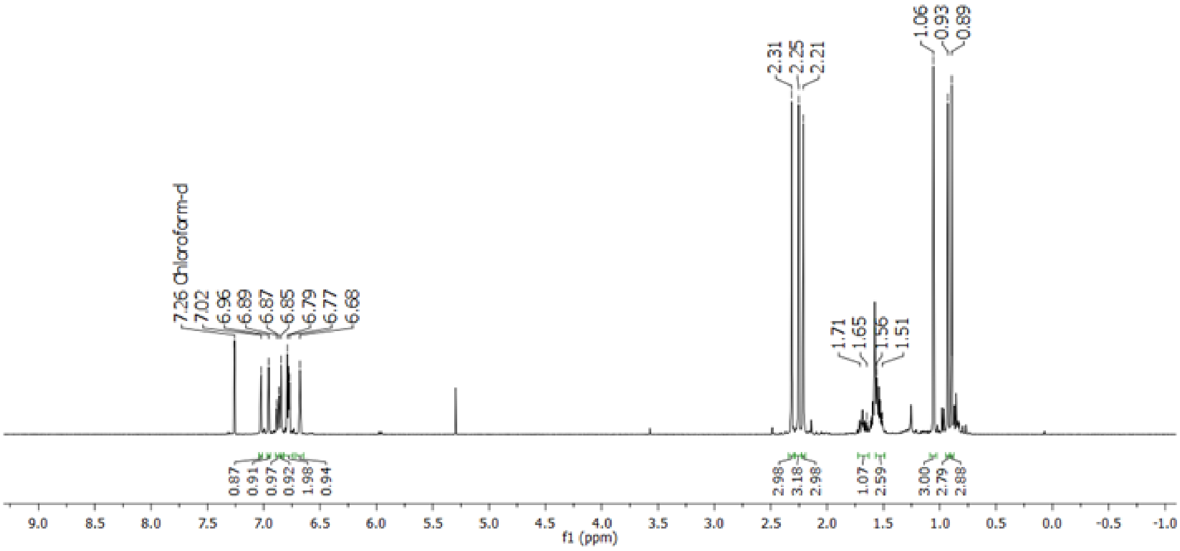

LM251-2diastero-F5-CDCl3-13CNMR-100MHz

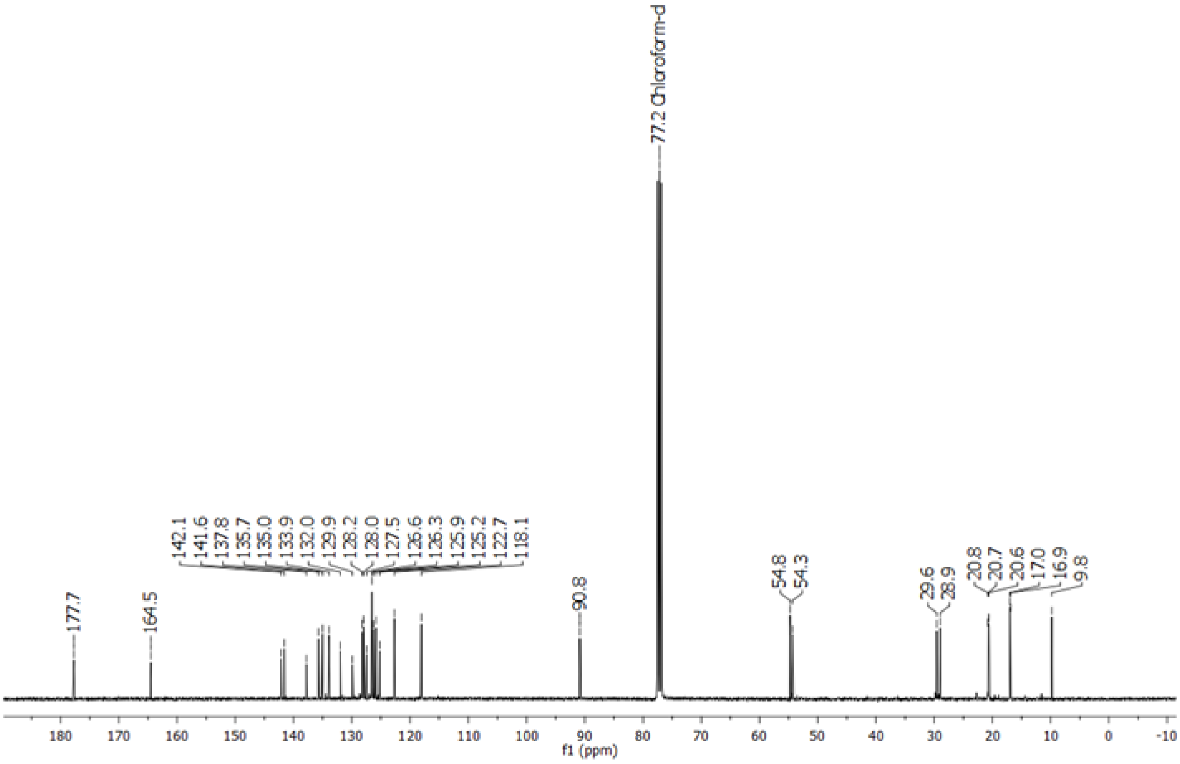

DFT calculations of compound 1b(OH)

The 3D-structures of compound **1b**(OH) in its *M* form have been optimized by DFT calculations and thereafter the CD and absorption spectra have been evaluated considering M06 and M06-2X functionals, the solvent has been treated at iefpcm level. Two conformers have been found with energy and population values reported in the following Table.

Table S1. Energy and Boltzmann population values for the two conformers reported in Figure 4 of the text

	Kcal/mole	pop	G Kcal/mole	pop
**M062X**				
**B**	1.38	*8.8%*	1.26	*10.6%*
**A**	0.00	*91.2%*	0.00	*89.4%*
**M06**				
**B**	1.06	*14.3%*	0.83	*19.7%*
**A**	0.00	*85.7%*	0.00	*80.3%*

Spectra have been simulated as superpositions of Gaussian-shaped bands, assuming 0.2 eV-bandwidths. The calculated average spectra are reported in Figure 5 in the text; below we report the calculated spectra of each conformer. A wavelength shift of +4 nm has been applied for the M06 calculation, of +26 nm for the M06-2X calculation: the applied shift has been chosen such as to maximize the similarity index S.I. introduced by as recommended in [Kuppens, T.; Langenaeker, W.; Tollenaere, J. P.; Bultinck, P. J. Phys. Chem. A 2003, 107, 542–553]. The similarity index obtained for the M06 calculated CD spectrum of Figure 5 in the text is 0.81 (0.96 for the absorption spectrum), the similarity index for the M062X CD spectrum is 0.76 (0.98 for the absorption spectrum).

CD and absorption experimental and calculated spectra with two choices of the DFT functional, both conformers are presented considering *M*-1b(OH).

Calculated spectra are presented for the two conformers A and B.

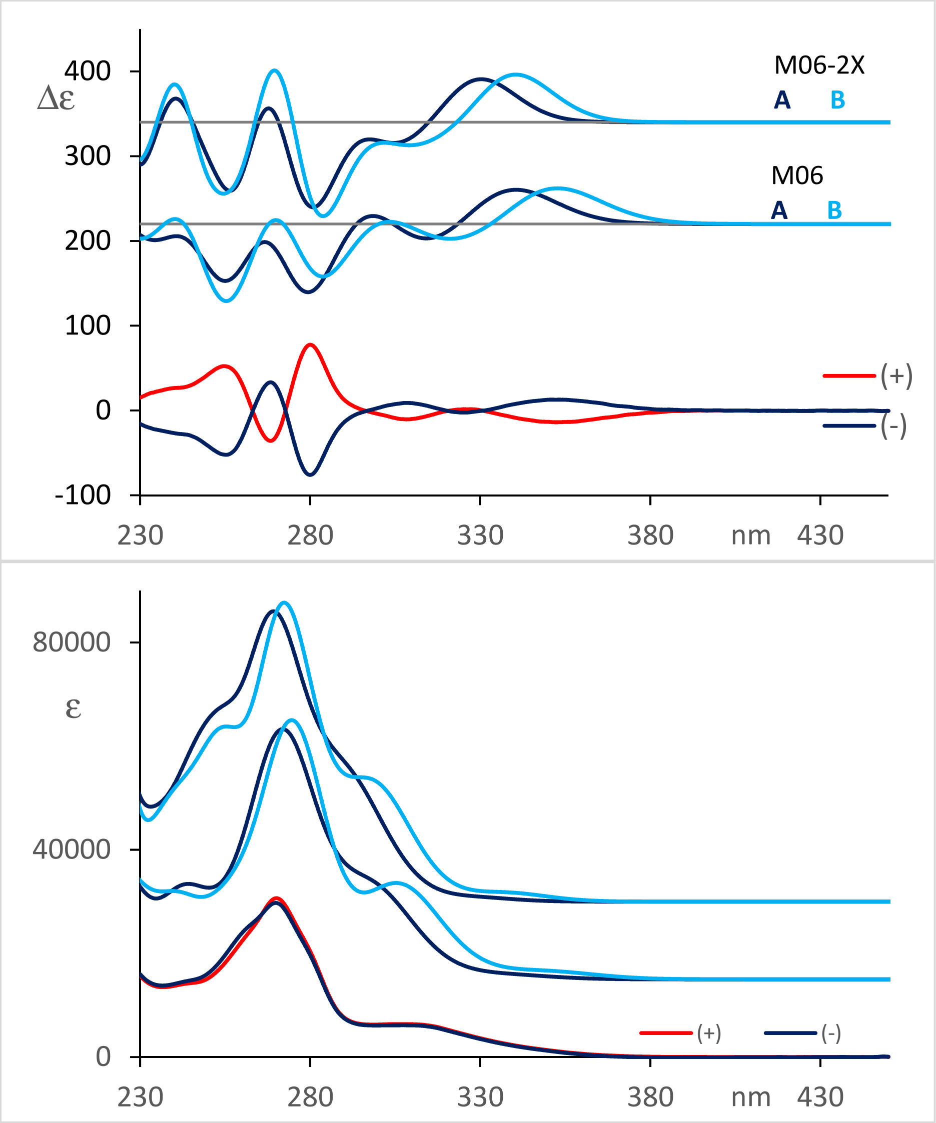

Optimized structures' coordinates.

Conformer A M06

Center Number	Atomic Number	Atomic Type	Coordinates (Angstroms) X	Y	Z
1	6	0	4.150720	-1.048962	0.283855
2	6	0	3.234180	-1.762657	1.042086
3	6	0	1.888342	-1.437703	1.028100
4	6	0	1.417702	-0.423713	0.186894
5	6	0	2.347584	0.320986	-0.525347
6	6	0	3.699366	0.022213	-0.475326
7	6	0	-0.918811	-1.114251	-0.184706
8	6	0	-0.620896	-2.190656	-1.008074
9	6	0	-2.207839	-0.995979	0.327951
10	6	0	-1.583581	1.592994	0.670678
11	6	0	-0.377619	1.215997	0.097420
12	6	0	0.370311	2.174936	-0.573569
13	1	0	4.402918	0.632165	-1.029937
14	6	0	-1.588767	-3.135915	-1.291380
15	6	0	-3.179824	-1.929123	0.009084
16	1	0	-4.182346	-1.804876	0.403627
17	6	0	-2.881358	-3.023988	-0.790769
18	7	0	0.037050	-0.129365	0.154547
19	6	0	-2.016457	2.907536	0.613133
20	6	0	-0.040726	3.497965	-0.588630
21	6	0	-1.235222	3.882688	0.007816
22	1	0	-2.973674	3.171929	1.047666
23	1	0	0.569458	4.236669	-1.096493
24	6	0	-1.665197	5.314093	0.000799
25	16	0	-2.567640	0.336456	1.449475
26	16	0	1.801616	1.694196	-1.505806
27	6	0	-3.915216	-4.060944	-1.090669
28	1	0	-3.783568	-4.473807	-2.091100
29	1	0	-3.851148	-4.894823	-0.387683
30	1	0	-4.922994	-3.652545	-1.018655
31	6	0	5.597802	-1.421440	0.297061
32	1	0	5.906716	-1.781575	1.278591
33	1	0	5.799375	-2.222695	-0.417628
34	1	0	6.229431	-0.576186	0.025421
35	1	0	-1.339247	-3.975045	-1.930210
36	1	0	0.376907	-2.284609	-1.419016
37	1	0	3.559895	-2.569173	1.688262
38	1	0	-2.750502	5.403223	0.042647
39	1	0	-1.261942	5.847154	0.864799
40	1	0	-1.311938	5.831129	-0.891275
41	8	0	1.061313	-2.132833	1.835604
42	1	0	0.192224	-1.713614	1.847202

Conformer B M06

Center Number	Atomic Number	Atomic Type	Coordinates (Angstroms) X	Y	Z
1	6	0	4.010156	-1.468163	0.320511
2	6	0	2.991695	-2.117694	1.006139
3	6	0	1.682415	-1.672140	0.946212
4	6	0	1.344250	-0.585596	0.129236
5	6	0	2.374404	0.078100	-0.523056
6	6	0	3.691828	-0.344083	-0.424048
7	6	0	-1.055886	-1.007372	-0.218435
8	6	0	-0.905389	-2.118249	-1.036629
9	6	0	-2.309698	-0.744956	0.322736
10	6	0	-1.387677	1.759879	0.641079
11	6	0	-0.241254	1.251040	0.044396
12	6	0	0.623025	2.136544	-0.587274
13	1	0	4.470123	0.211236	-0.934282
14	6	0	-1.974755	-2.958934	-1.278721
15	6	0	-3.385595	-1.572116	0.045624
16	1	0	-4.357066	-1.333402	0.465236
17	6	0	-3.232560	-2.703619	-0.743353
18	7	0	0.013229	-0.131849	0.050046
19	6	0	-1.653487	3.119448	0.622012
20	6	0	0.381881	3.500480	-0.560412
21	6	0	-0.759912	4.010763	0.043462
22	1	0	-2.568914	3.486573	1.072292
23	1	0	1.084833	4.172592	-1.040039
24	6	0	-1.013594	5.483379	0.079667
25	16	0	-2.508405	0.631926	1.428088
26	16	0	2.001901	1.505333	-1.508609
27	6	0	-4.381000	-3.626408	-0.997352
28	1	0	-4.321770	-4.068501	-1.992378
29	1	0	-4.391635	-4.450448	-0.279815
30	1	0	-5.336221	-3.108773	-0.910007
31	6	0	5.413980	-1.974026	0.397241
32	1	0	5.679666	-2.253961	1.417446
33	1	0	5.542668	-2.864600	-0.221967
34	1	0	6.126085	-1.226122	0.050746
35	1	0	-1.835262	-3.826521	-1.913448
36	1	0	0.063490	-2.322357	-1.476517
37	1	0	3.223617	-2.975297	1.630600
38	1	0	-2.081166	5.702584	0.091127
39	1	0	-0.580889	5.933271	0.976213
40	1	0	-0.569460	5.984562	-0.780258
41	8	0	0.692097	-2.253257	1.655350
42	1	0	1.049632	-2.985661	2.166051

Conformer A M06-2X

Center Number	Atomic Number	Atomic Type	Coordinates (Angstroms) X	Y	Z
1	6	0	4.186950	-0.915600	0.278361
2	6	0	3.291055	-1.676816	1.020656
3	6	0	1.934737	-1.390975	1.012277
4	6	0	1.432111	-0.367492	0.197243
5	6	0	2.338260	0.417905	-0.505006
6	6	0	3.701952	0.157605	-0.460153
7	6	0	-0.876373	-1.141189	-0.174951
8	6	0	-0.521342	-2.217366	-0.980342
9	6	0	-2.177355	-1.065548	0.320433
10	6	0	-1.654182	1.543694	0.661784
11	6	0	-0.424374	1.213810	0.104097
12	6	0	0.297758	2.201559	-0.559070
13	1	0	4.384354	0.796915	-1.004886
14	6	0	-1.451268	-3.202400	-1.269777
15	6	0	-3.111006	-2.039764	-0.005649
16	1	0	-4.121766	-1.949373	0.372604
17	6	0	-2.758338	-3.131433	-0.791924
18	7	0	0.039713	-0.118334	0.174555
19	6	0	-2.136571	2.843984	0.597628
20	6	0	-0.163524	3.510800	-0.577839
21	6	0	-1.382439	3.849766	0.003143
22	1	0	-3.107886	3.070462	1.018305
23	1	0	0.425150	4.271143	-1.075907
24	6	0	-1.867423	5.272657	-0.009030
25	16	0	-2.594623	0.253987	1.431082
26	16	0	1.750685	1.775331	-1.477185
27	6	0	-3.753629	-4.214994	-1.101154
28	1	0	-3.622736	-4.586506	-2.116518
29	1	0	-3.626177	-5.060650	-0.423224
30	1	0	-4.774647	-3.853700	-0.993854
31	6	0	5.653311	-1.246070	0.286937
32	1	0	5.984049	-1.530701	1.284829
33	1	0	5.859321	-2.085819	-0.378715
34	1	0	6.248567	-0.399359	-0.048762
35	1	0	-1.157921	-4.038370	-1.891930
36	1	0	0.486267	-2.280219	-1.369541
37	1	0	3.638502	-2.489227	1.645442
38	1	0	-2.953278	5.316575	0.050810
39	1	0	-1.464699	5.821851	0.843575
40	1	0	-1.547864	5.787521	-0.913521
41	8	0	1.122853	-2.137754	1.799773
42	1	0	0.243099	-1.742075	1.832899

Conformer B M06-2X

Center Number	Atomic Number	Atomic Type	Coordinates (Angstroms) X	Y	Z
1	6	0	4.069110	-1.319315	0.312972
2	6	0	3.071929	-2.025415	0.980000
3	6	0	1.746727	-1.622399	0.926189
4	6	0	1.371300	-0.524122	0.138196
5	6	0	2.377030	0.190210	-0.501834
6	6	0	3.711577	-0.188965	-0.407832
7	6	0	-1.008667	-1.046466	-0.207472
8	6	0	-0.796471	-2.158567	-1.013774
9	6	0	-2.280486	-0.831883	0.318703
10	6	0	-1.470738	1.707925	0.640948
11	6	0	-0.294310	1.250798	0.054795
12	6	0	0.541416	2.174739	-0.566352
13	1	0	4.468696	0.402915	-0.905263
14	6	0	-1.829479	-3.046314	-1.265402
15	6	0	-3.319141	-1.706607	0.030885
16	1	0	-4.303813	-1.506465	0.435660
17	6	0	-3.106976	-2.837530	-0.750459
18	7	0	0.020691	-0.122718	0.072204
19	6	0	-1.794181	3.058085	0.622409
20	6	0	0.241961	3.529919	-0.535759
21	6	0	-0.932371	3.989020	0.052952
22	1	0	-2.724945	3.384408	1.069022
23	1	0	0.925407	4.232432	-0.996307
24	6	0	-1.279096	5.452080	0.035282
25	16	0	-2.540469	0.532217	1.421334
26	16	0	1.951958	1.607687	-1.473891
27	6	0	-4.220582	-3.813363	-1.012908
28	1	0	-4.094678	-4.299243	-1.979245
29	1	0	-4.238923	-4.594654	-0.250914
30	1	0	-5.189654	-3.317223	-0.999434
31	6	0	5.498870	-1.777675	0.382302
32	1	0	5.773529	-2.047055	1.401619
33	1	0	5.649980	-2.660086	-0.241341
34	1	0	6.176486	-1.000624	0.034957
35	1	0	-1.642469	-3.912038	-1.888156
36	1	0	0.186860	-2.327364	-1.433107
37	1	0	3.331245	-2.890654	1.579863
38	1	0	-1.929394	5.710273	0.869039
39	1	0	-0.382538	6.067124	0.091510
40	1	0	-1.801331	5.712562	-0.886818
41	8	0	0.767278	-2.258482	1.614733
42	1	0	1.144116	-2.990827	2.113475
